# Gene Conversion Occurs within the Mating-Type Locus of *Cryptococcus neoformans* during Sexual Reproduction

**DOI:** 10.1371/journal.pgen.1002810

**Published:** 2012-07-05

**Authors:** Sheng Sun, Yen-Ping Hsueh, Joseph Heitman

**Affiliations:** Department of Molecular Genetics and Microbiology, Duke University Medical Center, Durham, North Carolina, United States of America; The University of North Carolina at Chapel Hill, United States of America

## Abstract

Meiotic recombination of sex chromosomes is thought to be repressed in organisms with heterogametic sex determination (e.g. mammalian X/Y chromosomes), due to extensive divergence and chromosomal rearrangements between the two chromosomes. However, proper segregation of sex chromosomes during meiosis requires crossing-over occurring within the pseudoautosomal regions (PAR). Recent studies reveal that recombination, in the form of gene conversion, is widely distributed within and may have played important roles in the evolution of some chromosomal regions within which recombination was thought to be repressed, such as the centromere cores of maize. *Cryptococcus neoformans*, a major human pathogenic fungus, has an unusually large mating-type locus (*MAT*, >100 kb), and the *MAT* alleles from the two opposite mating-types show extensive nucleotide sequence divergence and chromosomal rearrangements, mirroring characteristics of sex chromosomes. Meiotic recombination was assumed to be repressed within the *C. neoformans MAT* locus. A previous study identified recombination hot spots flanking the *C. neoformans MAT*, and these hot spots are associated with high GC content. Here, we investigated a GC-rich intergenic region located within the *MAT* locus of *C. neoformans* to establish if this region also exhibits unique recombination behavior during meiosis. Population genetics analysis of natural *C. neoformans* isolates revealed signals of homogenization spanning this GC-rich intergenic region within different *C. neoformans* lineages, consistent with a model in which gene conversion of this region during meiosis prevents it from diversifying within each lineage. By analyzing meiotic progeny from laboratory crosses, we found that meiotic recombination (gene conversion) occurs around the GC-rich intergenic region at a frequency equal to or greater than the meiotic recombination frequency observed in other genomic regions. We discuss the implications of these findings with regards to the possible functional and evolutionary importance of gene conversion within the *C. neoformans MAT* locus and, more generally, in fungi.

## Author Summary

Recombination has been thought to be repressed within sex chromosomes, as well as within the mating-type (*MAT*) loci in many fungi, due to the highly diverged and rearranged nature between alleles defining opposite sexes or mating-types. However, it has long been appreciated that recombination can occur within these presumptive recombinational “cold spots,” and recent studies reveal that recombination, including gene conversion, can occur at a frequency higher than previously appreciated and could play important roles in shaping evolution of these chromosomal regions. Here, we provide evidence that, during sexual reproduction of the human pathogenic fungus *Cryptococcus neoformans*, recombination (gene conversion) occurs across a GC-rich intergenic region within the *MAT* locus. The frequency of this gene conversion is comparable to those of typical meiotic recombination events observed in other chromosomal regions. This is in accord with population genetics analyses, which indicate homogenization between alleles of opposite mating-types within the intergenic region. Gene conversion within these highly rearranged chromosomal regions may serve to ensure proper meiosis and/or rejuvenate genes/chromosomal regions within *MAT* that are otherwise facing irreversible evolutionary decay. In conclusion, our study provides further experimental evidence that at least some recombinational “cold spots” are not that cold, after all.

## Introduction

During meiosis, recombination occurs to promote genetic exchange and ensure the proper segregation of homologous chromosomes. This process is initiated by the introduction of genome wide DNA double strand breaks (DSBs), followed by strand invasion and elongation. If the second end of the DSB is captured, a double Holliday Junction (double-HJ) will form. Resolving of the resulting double-HJ usually results in exchange of genetic information between the two homologous chromosomes. Depending on the way that the double-HJ is resolved (i.e. resolution or dissolution), this exchange can be either reciprocal (i.e. crossing-over), or unidirectional (i.e. gene conversion). It should be noted that although all recombination events are accompanied by gene conversion, only a fraction of recombination events actually result in crossing-over [1], [2], [3],[4],[5]. Alternatively, after the initial strand invasion and elongation, DSBs can also be repaired through a synthesis-dependent strand-annealing (SDSA) pathway, which only produces gene conversion [6], [7].

Studies have shown there are homeostatic controls acting on crossover formation during meiosis in both yeast and mouse [8], [9]. Additionally, recombination frequency is not evenly distributed across genomes, with certain regions being “hot spots”, where recombination occurs at higher frequencies, and other regions being “cold spots” that experience recombination at comparatively lower frequencies [10]. Some examples of recombination “hot spots” include the major histocompatibility complex (MHC) in humans [11], [12], as well as the regions flanking the *MAT* locus in the human pathogenic fungus *Cryptococcus neoformans* [13]. On the other hand, it has been shown that in some areas of the genome, such as centromeric regions and sex chromosomes, recombination is repressed during meiosis, largely due to the existence of repetitive sequences and extensive chromosomal rearrangements within these regions that prevent the proper pairing between the homologous chromosomes during meiosis. However, a recent study by Shi et al. reported that recombination, in the form of gene conversion, is widespread within the centromeric regions of maize [14], and studies have shown that meiotic recombination can occur within the less complex yeast centromere [15]. Additionally, gene conversion has also been reported to occur within human and ape Y chromosomes, as well as between X-Y homologues located within the nonrecombining region of the Y chromosome in Felidae [16], [17], [18]. Nevertheless, recombination frequencies within these presumed “cold spots” are still considered to be much lower than those within typical chromosomal regions.

Recombination frequencies have been shown to be positively correlated with the local GC content in a variety of species, including humans [19], [20], the yeast *Saccharomyces cerevisiae* [21], and the human pathogenic fungus *C. neoformans* [13]. Several studies support the view that recombinational activity is driving the local GC composition [22], [23], [24], [25], [26], possibly through a process in which recombination-associated gene conversion introduces a bias in favor of GC, also called GC-biased gene conversion (gBGC) [27], thus increasing the local GC content around the recombination hot spots. However, a recent study of the yeast *S. cerevisiae* suggests that local GC content is not driven by recombination [21]. Additionally, the local GC content may also influence the susceptibility of a given chromosomal region to mutagenic factors, such as UV radiation [28], and thus indirectly affect the local frequency of recombination required for DNA damage repair processes.

Sex chromosomes, such as those in mammals and birds, and larger mating type (*MAT*) loci of several fungi (e.g. the *MAT* locus of *C. neoformans*), usually have extensive sequence divergence and chromosome rearrangements between opposite alleles, and are thus thought to be suppressed for recombination during meiosis. This repression of recombination protects the integrity of the linked sex determining genes, and prevents generation of deleterious abnormal chromosomes resulting from recombination within rearranged chromosomal regions. For organisms with heterogametic sex, the proper segregation of the sex chromosomes is usually ensured by recombination occurring within a defined region within the sex chromosomes, such as the pseudo-autosomal region (PAR) of the mammalian sex chromosomes. Studies have shown that crossing-over occurring within these regions is crucial for the proper segregation of the two sex chromosomes during meiosis [29]. Similarly, recombinational hot spots have also been identified flanking the non-recombining *MAT* locus in *C. neoformans* [13]. Despite the facts that recombination within the PAR maintains homology between the opposite sex chromosomes [30], as well as that certain selection processes, such as purifying selection, operate to ensure the proper function of these regions, the non-recombining nature of the majority of the sex chromosomes, as well as the fungal *MAT* loci, threatens these regions with gradual deterioration, as suggested for the mammalian Y chromosomes [31].

A previous study by Hsueh et al. [13] showed that recombination hot spots exist in the regions flanking the *MAT* locus of the human pathogenic fungus *C. neoformans*, and these regions have an unusually high GC content. In the same study, a minor GC peak was identified that is located within the intergenic region of the *RPO41* and *BSP2* genes in the *MAT* locus. However, whether or not this minor GC peak is also associated with a higher recombination frequency was not analyzed due to the lack of polymorphic markers within this region. The objective of this study was to investigate whether this minor GC rich region also shows unique features in recombination frequency during meiosis. To address recombination in this GC rich region, we employed both population genetics analyses of natural isolates, as well as analysis of meiotic progeny generated from laboratory crosses. We found that recombination, in the form of gene conversion, occurs at a frequency that is at least comparable to the meiotic recombination frequencies in other chromosomal regions, and this observation is also supported by results from population genetic analyses showing homogenization spanning this intergenic GC rich region in natural strains. These observations have implications for the evolution of mating type loci and sex chromosomes, and the nature and formation of recombination in other complex genomic regions, such as centromeres.

## Results

### The GC-rich intergenic region between the *RPO41* and *BSP2* genes is present in all lineages of *C. neoformans* and *C. gattii*

**Figure 1:**
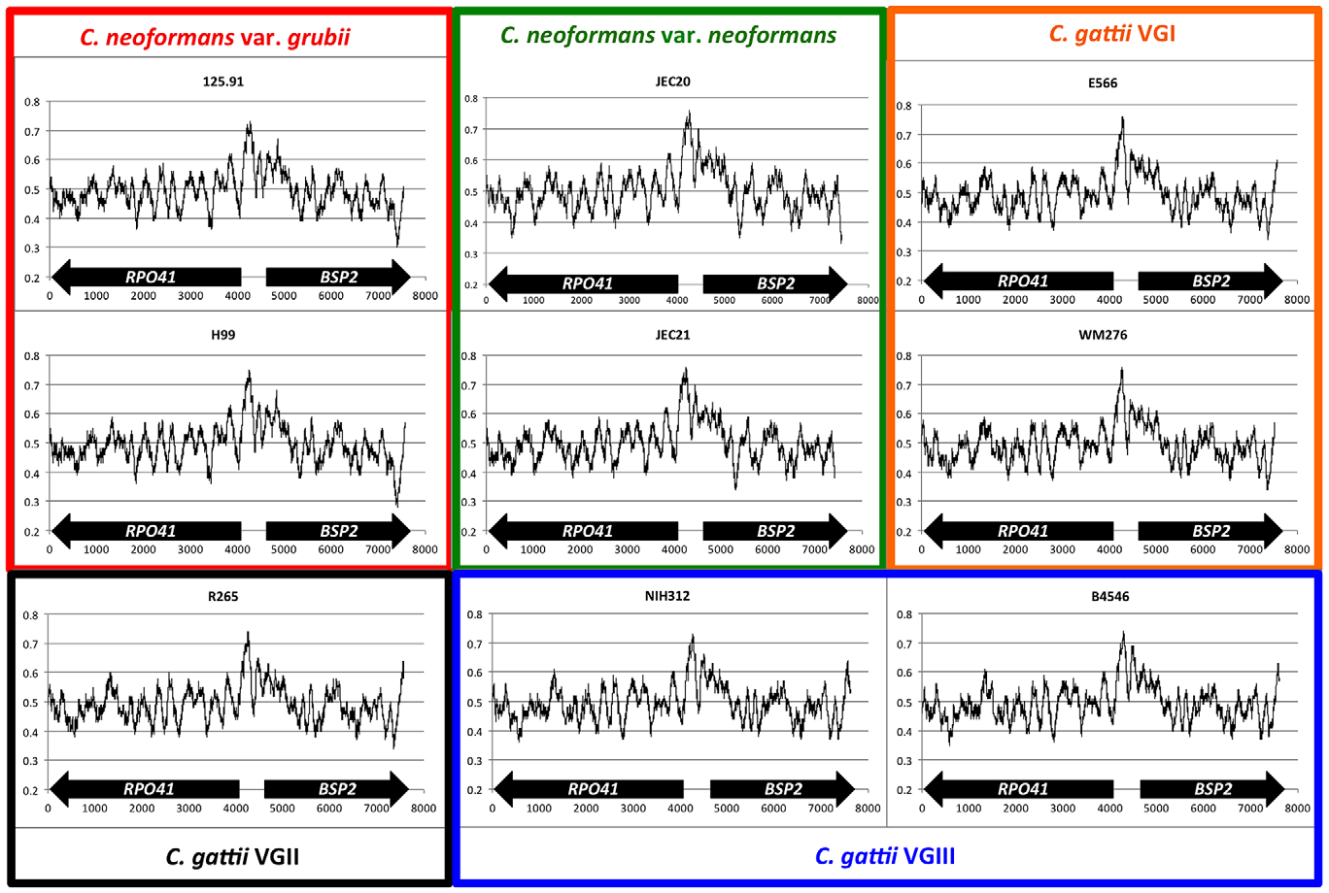
Sliding window analyses of GC content of the regions encompassing *RPO41*, intergenic region, and *BSP2* in *C. neoformans* and *C. gattii*. For each figure, the Y axis is the GC%, while the X axis indicates the relative distance (bp) from the 3′ end of the *RPO41* gene. Sequences from the *MAT* **a** and *MAT*α reference strains of the same variety (as in *C. neoformans*) or the same molecular group (as in *C. gattii*) are grouped together by rectangles of different colors. The size of the sliding window was 200 bp.

The GC rich intergenic region between the *RPO41* and *BSP2* genes in the *MAT* locus of *C. neoformans* var. *neoformans* was first identified by Hsueh et al [13]. Taking advantage of the existing genomic sequences of species of the pathogenic *Cryptococcus* species complex [32], [33], we further investigated whether this GC rich region is present in other lineages that are closely related to *C. neoformans*. GC plots of the homologous regions from strains 125.91 (*C. neoformans* var. *grubii*, *MAT* **a**), H99 (*C. neoformans* var. *grubii*, *MAT*α), E566 (*Cryptococcus gattii*, VGI, *MAT* **a**), WM276 (*C. gattii*, VGI, *MAT*α), R265 (*C. gattii*, VGII, *MAT*α), NIH312 (*C. gattii*, VGIII, *MAT* **a**), and B4546 (*C. gattii*, VGIII, *MAT*α) all showed GC peaks at similar positions (Figure 1), reflecting the common origin of this region in these lineages. It also suggests this GC rich intergenic region may be functionally important, such that the high GC content is being maintained through either natural selection (e.g. purifying selection) or other molecular mechanisms (e.g. gene conversion).

### Population genetic analyses revealed homogenization between *MAT*a and *MAT*α alleles around the GC-rich intergenic region in natural *C. neoformans* isolates

**Figure 2:**
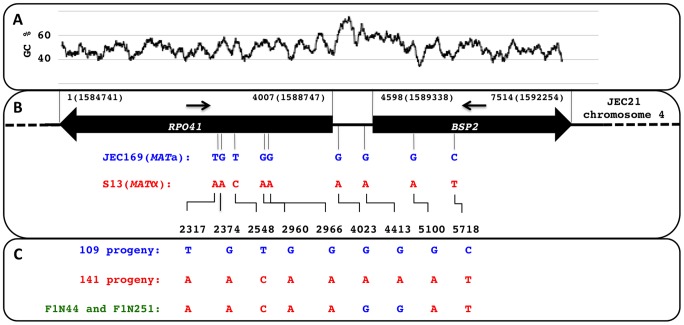
Distribution of polymorphic sites between the two parental strains and the genotypes identified among the meiotic progeny. (A) Illustration of the distribution of GC content along the region encompassing the *RPO41* and *BSP2* genes, as well as the intergenic region between the two genes. (B) Positions of the nine polymorphic sites present within the sequenced region between the two strains, JEC169 (*MAT* **a**) and S13 (*MAT*α), which were used in the laboratory cross to generate meiotic progeny. The two arrows indicate the approximate locations of the forward and reverse primers used to amplify this region from both natural isolates and progeny generated by laboratory cross. (C) Summary of the genotypes identified among 255 meiotic progeny based on the above mentioned nine polymorphic sites.

We sequenced a region that encompasses the 5′ ends of the *RPO41* and *BSP2* genes, and the GC rich intergenic region between the *RPO41* and *BSP2* genes from a group of natural *C. neoformans* strains, including both var. *grubii* and var. *neoformans* (Table 1 and Figure 2). We also PCR amplified and sequenced serotype A and serotype D specific alleles from a group of AD hybrid isolates (Table 1 and Table S1).

**Table 1:** List of strains used in this study.

Strain Name	Serotype	Mating type^a^	VN type^a^
JEC169^b^	D	**a**	VNIV
S13^c^	D	α	VNIV
Bt204	A	**a**	VNB
Bt206	A	**a**	VNB
Bt24	A	**a**	VNB
Bt63	A	**a**	VNB
Bt65	A	**a**	VNB
Bt85	A	**a**	VNB
Bt88	A	**a**	VNB
Bt109	A	α	VNB
Bt125	A	α	VNB
Bt22	A	α	VNB
Bt31	A	α	VNB
Bt33	A	α	VNB
Bt34	A	α	VNB
Bt60	A	α	VNB
Bt84	A	α	VNB
Bt89	A	α	VNB
125.91	A	**a**	VNI
Bt130	A	**a**	VNI
Bt104	A	α	VNI
Bt121	A	α	VNI
Bt134	A	α	VNI
Bt15	A	α	VNI
Bt150	A	α	VNI
Bt57	A	α	VNI
Bt68	A	α	VNI
H99	A	α	VNI
KN99**a**	A	**a**	VNI
KN99alpha	A	α	VNI
CDC94-383	AD	-AD**a**	VNIII
ATCC48184	AD	-ADα	VNIII
CDC228	AD	**a**ADα	VNIII
CDC304	AD	**a**ADα	VNIII
CDC92-74	AD	**a**ADα	VNIII
IT752	AD	**a**ADα	VNIII
IUM92-6198	AD	**a**ADα	VNIII
MMRL752	AD	**a**ADα	VNIII
NC34-21	AD	**a**ADα	VNIII
ZG290	AD	**a**ADα	VNIII
CBS132	AD	αAD**a**	VNIII
KW5	AD	αAD**a**	VNIII
MMRL774	AD	αAD**a**	VNIII
ZG287	AD	αAD**a**	VNIII
5-19	AD	αADα	VNIII
713	AD	αADα	VNIII
JEC20	D	**a**	VNIV
NIH264	D	**a**	VNIV
NIH276	D	**a**	VNIV
NIH430	D	**a**	VNIV
NIH433	D	**a**	VNIV
431	D	α	VNIV
434	D	α	VNIV
528	D	α	VNIV
529	D	α	VNIV
709	D	α	VNIV
710	D	α	VNIV
711	D	α	VNIV
B3179	D	α	VNIV
CAP67-2	D	α	VNIV
CDC92-18	D	α	VNIV
CDC92-27	D	α	VNIV
J9	D	α	VNIV
JEC21	D	α	VNIV
MMRL751	D	α	VNIV
MMRL757	D	α	VNIV
MMRL760	D	α	VNIV
NIH12	D	α	VNIV
S14	D	α	VNIV
S15	D	α	VNIV
VANC.R461	D	α	VNIV
Y290-90	D	α	VNIV

a : Determined in previous studies [51], [52], [53].

b : *ade2 ura5*; one of the two parental strains used in the laboratory cross.

c : *ADE2 URA5*; one of the two parental strains used in the laboratory cross; also included in the population genetics analyses of natural isolates.

**Figure 3:**
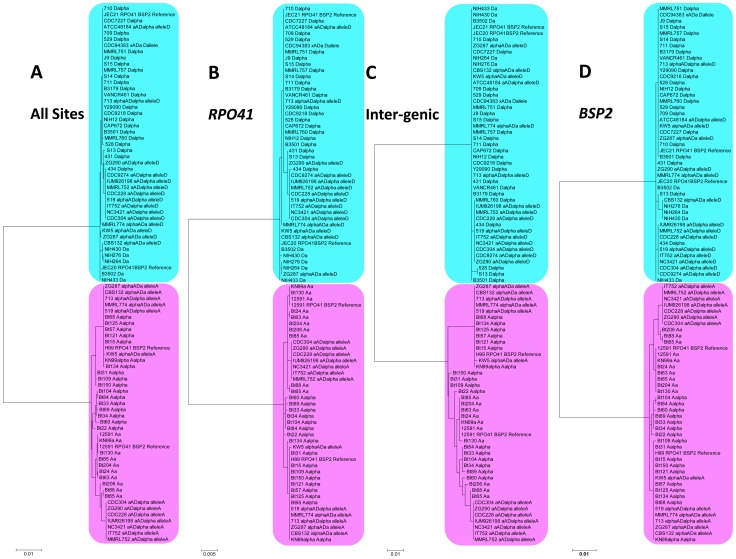
Phylogenetic trees of alleles existing in natural *C. neoformans* isolates based on the sequenced *RPO41*-intergenic-*BSP2* region. Phylogenetic trees were constructed using maximum likelihood methods with 1000 bootstraps. Alleles shaded with pink color were all serotype A (from both haploid and hybrid isolates), while alleles shaded with blue color were all serotype D (from both haploid and hybrid isolates). A) Sequences from the entire *RPO41*-intergenic-*BSP2* region were analyzed. B) Only sequences within the gene *RPO41* were used. C) Only sequences within the intergenic region were studied. D) Only sequences within the gene *BSP2* were subjected to analysis.

Overall, this region showed a serotype specific phylogeny when all of the alleles are compared together, as all of the serotype A alleles (from both haploid var. *grubii* isolates and serotype AD hybrids) grouped within a well supported cluster that showed considerable divergence from the cluster that included all of the serotype D alleles (Figure 3). This pattern still held when the regions belonging to the three sections (the two genes, *RPO41* and *BSP2*, and the intergenic regions) were analyzed separately (Figure 3), consistent with the view that serotypes A and D are well separated lineages that diverged from each other long ago. Among the three sections, the intergenic region showed the highest level of divergence between the two clusters of serotype A and serotype D alleles (Figure 3). Within each cluster, the level of polymorphism within the intergenic region is comparable to the other two gene coding regions among serotype D alleles, and is higher than the other two sections among serotype A alleles (Table 2). No signal of positive selection (d_N_/d_S_>1) was detected in either *RPO41* or *BSP2* gene coding region for either serotype A or serotype D alleles.

**Table 2:** Genetic distances of different genes/regions within the *MAT* locus.^a^

	Region	Over all mean distance
Gene	*RPO41* b	0.001 (0.002)^c^
	*BSP2* b	0.001 (0.004)^c^
	*IKS1* d	0.005
	*NCM1* d	0.007
	*SXI1* d	0.005
Inter-genic region	*RPO41* – *BSP2* b	0.001 (0.012)^c^
	*IKS1* – *CND05650* d	0.01
	*PRT1* – *ZNF1* d	0.001
	*RPO41* – *STE12* d	0.003

a : calculated using program MEGA 5; the genetic distances listed in the table represent estimates of the genetic differences for *C. neoformans* var. *neoformans* (serotype D) isolates unless indicated otherwise;

b : calculated from both the *MAT* **a** and *MAT*α alleles;

c : numbers in parentheses are for *C. neoformans* var. *grubii* (serotype A) alleles;

d : calculated from only the *MAT*α alleles.

**Figure 4:**
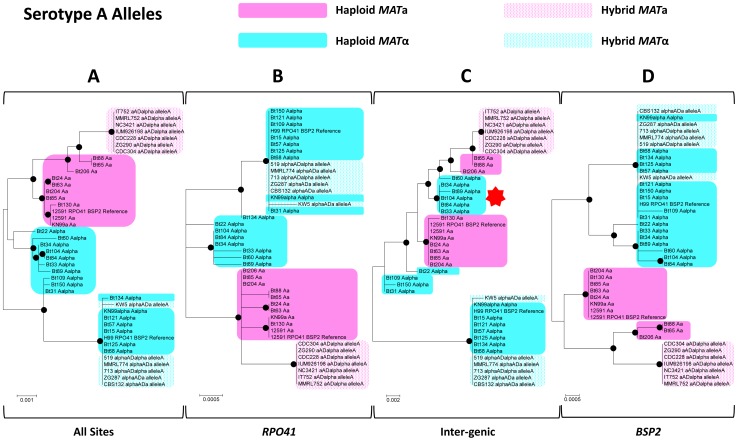
Phylogenetic trees of serotype A alleles existing in natural *C. neoformans* isolates based on the sequenced *RPO41*-intergenic-*BSP2* region. Phylogenetic trees based on whole sequences (A), sequences corresponding to the *RPO41* gene (B), the intergenic regions (C), and the *BSP2* gene (D) were constructed using the maximum likelihood method with 1000 bootstrap replications. Black dots indicate the nodes supported by bootstrap values of 75 or higher. Solid and dotted pink shades highlight *MAT* **a** alleles from haploid and hybrid isolates, respectively. Solid and dotted blue shades highlight *MAT*α alleles from haploid and hybrid isolates, respectively. The red star in section (C) highlights the group of haploid *MAT*α alleles that clustered together with *MAT* **a** alleles.

When only serotype A alleles were considered, well supported clusters containing all and only the *MAT* **a** specific alleles were observed when all of the sites were included in the analyses (Figure 4A), as well as when the regions corresponding to the two genic regions were analyzed (Figure 4B and 4D). However, when only the intergenic region was analyzed, this mating type specific topology no longer held. Instead, *MAT*α alleles from six haploid strains were placed within a well supported cluster that otherwise contained only *MAT* **a** specific alleles (Figure 4C). The phylogeny of the intergenic region was shown to be statistically different (p<0.01) from those of the two flanking genes by the Shimodaira-Hasegawa test [34].

**Figure 5:**
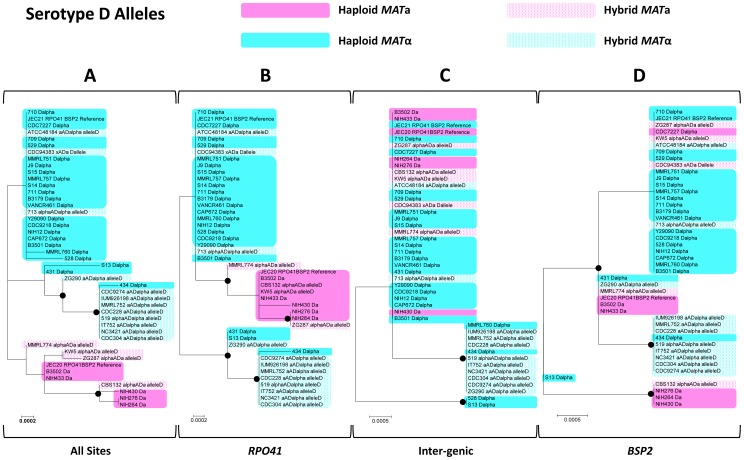
Phylogenetic trees of serotype D alleles existing in natural *C. neoformans* isolates based on the sequenced *RPO41*-intergenic-*BSP2* region. Phylogenetic trees based on whole sequences (A), sequences corresponding to the *RPO41* gene (B), the intergenic regions (C), and the *BSP2* gene (D) were constructed using the maximum likelihood method with 1000 bootstrap replications. Black dots indicate the nodes supported by bootstrap values of 75 or higher. As in Figure 4, solid and dotted pink shades highlight *MAT* **a** alleles from haploid and hybrid isolates, respectively, while solid and dotted blue shades highlight *MAT*α alleles from haploid and hybrid isolates, respectively.

**Figure 6:**
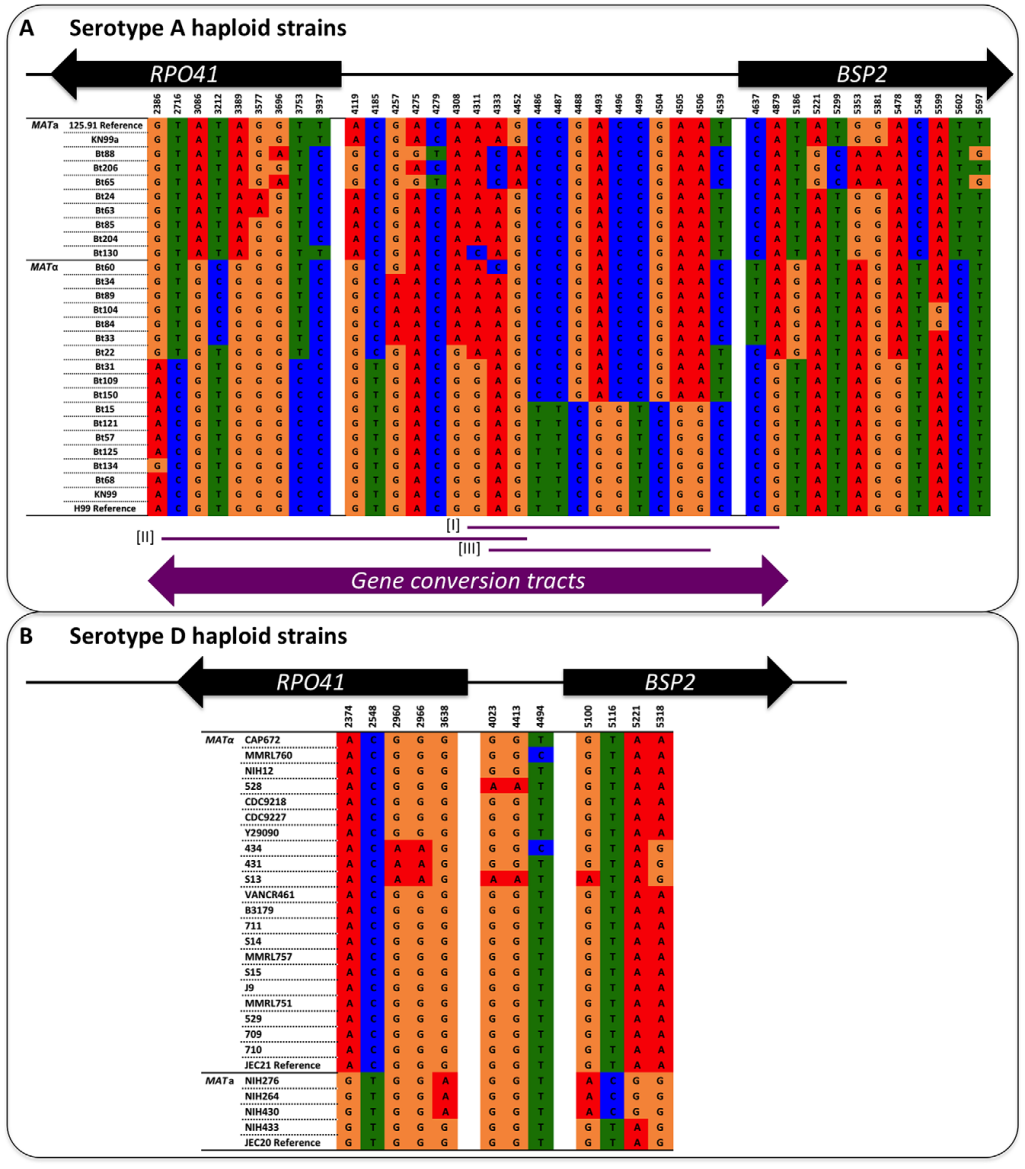
Illustration of polymorphic sites among serotype A and D haploid strains. The positions of the polymorphic sites are shown at the top, and they correspond to the distances of the sites from the 3′ end of the *RPO41* gene (see also Figure 2). For simplicity, only parsimony informative sites are shown here. (A) Serotype A strains. Sites from 2386 to 3937 are located within the *RPO41* gene, sites from 4119 to 4539 are located within the intergenic region, while sites from 4637 to 5697 are located within the *BSP2* gene. The purple lines and arrow at the bottom indicate locations and lengths of gene conversion tracts detected by GENECONV software. Examples of strain pairs from which the three gene conversion tracts were detected are: Bt22-Bt150 for tract [I], Bt109-Bt125 for tract [II], and Bt60-Bt31 for tract [III]. (B) Serotype D strains. Sites from 2374 to 3638 are located within the *RPO41* gene, sites from 4023 to 4494 are located within the intergenic region, while sites from 5100 to 5318 are located within the *BSP2* gene.

When only serotype D alleles were considered, again a well supported cluster containing all and only the *MAT* **a** specific alleles was observed when all of the sites were analyzed (Figure 5A), as well as when the region corresponding to the gene *RPO41* was analyzed separately (Figure 5B). When the regions corresponding to the intergenic region and the *BSP2* gene were analyzed separately, this mating type specific phylogeny was no longer well supported. Specifically, when only the region within the *BSP2* gene was analyzed, four *MAT* **a** specific alleles formed a well supported cluster, while the other five *MAT* **a** specific alleles were grouped together with most of the *MAT*α specific alleles within a well supported cluster, reflecting a sharing of polymorphisms between alleles from the two mating types in this region. For the intergenic region, other than the two well supported clusters, one containing two *MAT*α haploid alleles and the other containing two haploid *MAT*α alleles and nine *MAT*α alleles from hybrid AD isolates, all of the other *MAT* **a** and *MAT*α alleles grouped together, indicating a lack of divergence between *MAT* **a** and *MAT*α alleles at the GC rich intergenic region (Figure 6). Similar to serotype A alleles, the topology of the intergenic region was shown to be statistically different (p<0.01) from those of the *RPO41* and *BSP2* genes by the Shimodaira-Hasegawa test [34].

We further looked for possible gene conversion tracts in this region in the natural population of *C. neoformans*. For serotype D alleles, we did not detect any statistically significant gene conversion tracts, possibly due to the low level of polymorphism present around the GC rich intergenic region. However, when serotype A alleles were analyzed, we indeed found several statistically significant gene conversion tracts encompassing the GC rich intergenic region (Figure 6; p<0.05 based on 10000 permutations). The lengths of the gene conversion tracts ranged from 560 to 2100 bp, consistent with previous studies in yeast showing most gene conversion tracts were between 1800 to 2000 bp [35]. These gene conversion tracts collectively spanned a 2.5 kb region encompassing the GC rich intergenic region (Figure 6).

Taken together, our population genetic analyses revealed that: 1) compared to the two genic regions, there was higher divergence between serotypes A and D alleles in the intergenic regions; 2) within serotype D alleles, the intergenic region showed a low level of polymorphisms compared to the flanking genic regions, as well as other intergenic regions within the *MAT* locus, even though no evidence of purifying selection was detected in the flanking genic regions of the *RPO41* and *BSP2* genes; 3) within serotype A and serotype D alleles, respectively, a mating type specific topology was observed for regions corresponding to the *RPO41* and *BSP2* genes, albeit the pattern was less well resolved for the serotype D *BSP2* region; 4) there was no mating type specific topology at the intergenic regions for both serotypes A and D alleles, and the phylogeny of this GC rich intergenic region is statistically different from those of the flanking genic regions of *RPO41* and *BSP2*, suggesting sequence exchange and homogenization of this GC rich intergenic region in both lineages; and 5) statistically significant gene conversion tracts were detected in serotype A alleles across the GC rich intergenic region. These observed patterns could be explained by a process of ongoing gene conversion at the GC rich intergenic region within each lineage to prevent divergence between the two mating types, while allowing polymorphisms to accumulate independently between the two serotypes.

### Meiotic gene conversion occurs around the GC peak within the *MAT* locus

We hypothesized that if gene conversion at the GC rich intergenic region occurs at a frequency high enough to produce the population structure observed among the natural isolates, we should be able to detect it in the meiotic progeny generated from laboratory crosses. To test this hypothesis, we crossed two fertile serotype D strains of opposite mating type, JEC169 (*MAT* **a**) and S13 (*MAT*α) and collected 260 recombinant progeny (i.e. the progeny whose phenotype with respect to auxotrophic mutations differed with both parental strains) by screening progeny for auxotrophic mutations present in the two parental strains (see Materials and Methods). We then PCR amplified and sequenced from each of these meiotic progeny the same region analyzed for the natural isolates and looked for gene conversion events that might have occurred around the intergenic region during meiosis.

Among these 260 recombinant progeny recovered, 259 of them had the recombinant genotype *ADE2 ura5*, while the other one was *ade2 URA5*. Additionally five of the recombinant progeny were filamentous when grown on YPD solid medium. Further analysis by FACS showed that these five progeny were diploid (data not shown). Thus, these five progeny were likely diploid fusion products of the two parental strains that underwent loss of heterozygosity at the auxotrophic markers (rather than haploid meiotic progeny), and consequently they were excluded from the following analyses.

The remaining 255 recombinant progeny grew as yeast (i.e. no filamentation) on YPD solid medium and our analyses indicated that they were haploid. For chromosome 4, where *MAT* is located, only the **a** or α allele was present, but not both. Mating assays by backcrossing each of these 255 progeny to the two parental strains confirmed that the majority are fertile. Specifically, 92 (36.1%) progeny typed as *MAT* **a** (i.e. successful mating with S13 but not JEC169); 139 (54.5%) progeny typed as *MAT*α (i.e. successful mating with JEC169 but not S13); and 24 (9.4%) progeny were sterile (i.e. no mating was observed with either parental strain).

The *RPO41-BSP2* intergenic region of these 255 F1 progeny was then PCR amplified and sequenced. Based on the polymorphic sites within this region between the two parental strains (Figure 2), 109 progeny inherited the alleles from the *MAT* **a** parent (strain JEC169), and 141 progeny inherited the alleles from the *MAT*α parent (strain S13). These genotypes are in accord with the mating phenotypes of the progeny determined by mating assays (see above). However, two progeny, F1N44 and F1N251, showed evidence of gene conversion at the *RPO41-BSP2* intergenic region (Figure 2). Specifically, these two isolates inherited alleles from JEC169 (*MAT* **a**) at the two markers located within the intergenic region (sites 4023 and 4413, Figure 2), and both inherited alleles from S13 (*MAT*α) at the loci located within the *RPO41* and *BSP2* genes. The most parsimonious explanation is that gene conversion events occurred across the GC rich intergenic region (Figure 2). In both cases, the two polymorphic sites within the intergenic regions were converted from A to G, consistent with the biased gene conversion in favor of G/C that has been reported previously [27]. Our analyses using PCR-RFLP markers located on other regions of chromosome 4 suggests these two isolates arose from independent gene conversion events, as they inherited different alleles at other markers (Table 3 and Table S2; also see below). Additionally, these two progeny still mate as *MAT*α, suggesting the conversion of the intergenic region from *MAT*α to *MAT* **a** does not have direct effects on the mating phenotype.

**Table 3:** Genotypes of the two parental strains and representative recombinant progeny at the 18 markers screened in this study.

	Phenotypic Markers			Polymorphic sites across *RPO41* and *BSP2* genes								PCR-RFLP markers located at other genes on chromosome 4^e^								
Isolate	Mating Type^a^	ADE^b^	URA^b^	2317^c^	2374^c^	2548^c^	2960^c^	2966^c^	4023^d^	4413^d^	5100^e^	CND03670^f^	CND03960^f^	CND04120^f^	CND04340^f^	CND04540^f^	CND05140^f^	CND05310^f^	SXI1/SXI2^g^	Number of crossing-overs between the *CND03670* gene and the *SXI1/SXI2* gene
JEC169	**a**	−	−	T	G	T	G	G	G	G	G	a	a	a	a	a	a	a	a	n.a.
S13	α	+	+	A	A	C	A	A	A	A	A	b	b	b	b	b	b	b	b	n.a.
F1N044^h^	α	+	−	A	A	C	A	A	G^I^	G^I^	A	a	a	a	a	a	a	b	b	1
F1N072^J^	α	−J	+^J^	A	A	C	A	A	A	A	A	b	b	a	a	a	b	b	b	2
F1N251	α	+	−	A	A	C	A	A	G^I^	G^I^	A	a	a	a	a	a	a	a	b	1

This table only includes the two parent strains and three highlighted unique meiotic progeny (see below). For the list including all recombinant progeny please see Table S2.

a : Mating types were determined based on mating assay by backcrossing each progeny to the two parental strains. “**a**” indicates *MAT* **a** progeny; “α” indicates *MAT*α progeny; “n.a.” indicates the progeny did not mate with either parent (i.e. the progeny is sterile).

b : “+” indicates wild type; “−” indicates auxtrophic;

c : polymorphic sites located within the *RPO41* gene;

d : polymorphic sites located within the inter-genic region between the *RPO41* and *BSP2* genes;

e : polymorphic sites located within the *BSP2* gene;

f : PCR-RFLP markers, see Table S1;

g : PCR markers, see Table S1;

h : “F1” indicates the strain was isolated from the F1 generation of the laboratory crosses between JEC169 and S13; “N044” indicates the strain is the progeny No. 44 in the series.

I : The nucleotides that underwent gene conversion in the two progeny, F1N044 and F1N251.

J : The only *ade2 URA5* progeny, F1N072.

Assuming DSBs occur only within the intergenic region, the maximum frequency of this gene conversion around the GC rich intergenic region could be estimated to be:
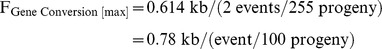
In which 0.614 kb is the size of the intergenic region. Alternatively, using the distance between the two polymorphic sites flanking the intergenic region, the minimum gene conversion frequency could be estimated as:
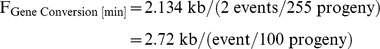
in which 2.134 kb is the distance between sites 2966 and 5100 in Figure 2.

### The frequency of gene conversion at the GC-rich intergenic region was at least comparable to the meiotic recombination frequencies elsewhere on the same chromosome

To put the observed frequency of gene conversion around the GC rich intergenic region into perspective, we calculated meiotic recombination frequencies at other chromosomal regions by constructing a genetic linkage map using the same meiotic progeny population and markers located on the same chromosome, chromosome 4, as the *MAT* locus. We then compared these meiotic recombination frequencies directly with the gene conversion frequency observed involving the GC rich intergenic region.

**Figure 7:**
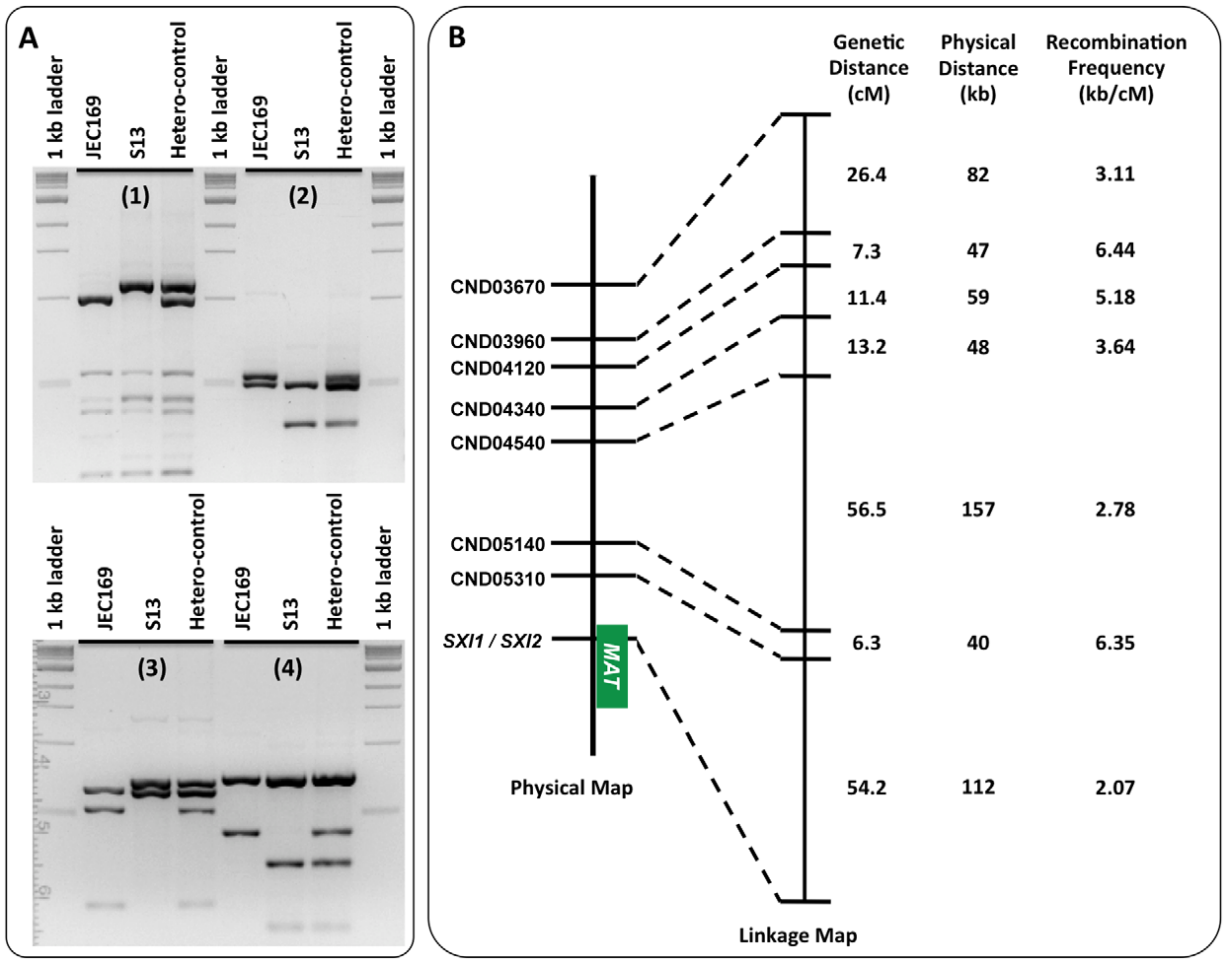
Meiotic recombination frequencies in chromosomal regions proximal to the *MAT* locus. (A) Examples of the co-dominant PCR-RFLP markers used to construct the genetic linkage map of the meiotic progeny shown in Figure 7B. Shown here are the gel separations of the restriction enzyme digestion products of markers CND05310 (A1), CND04540 (A2), CND03960 (A3), and CND04340 (A4). For each marker, from left to right, are the digestions of PCR products using genomic DNA of JEC169, S13, and a mixture of JEC169 and S13 (heterozygous control) as templates, respectively. (B) On the left is the physical map of the eight genetic markers on strain JEC20/JEC21 chromosome 4. The green block indicates the position of the *MAT* locus. In the middle is the genetic linkage map constructed using the PCR-RFLP data listed in Table 3 and Table S2. The three columns on the right list the genetic distances between adjacent markers, the physical distances between adjacent markers, and the corresponding recombination frequencies (high kb/cM value indicates low recombination frequency).

Specifically, we identified eight PCR/PCR-RFLP markers between the two parental strains (Table 3 and Table S2) that were located between ∼100 kb and ∼540 kb away from the *SXI1*α gene located at one end of the *MAT* locus. We then used these markers to screen all of the recombinant progeny. Five of the eight markers were co-dominant PCR-RFLP markers (Figure 7A), and we did not observe heterozygosity in any of the 255 recombinant progeny at any of these five markers, further corroborating that these isolates are haploid for chromosome 4.

**Figure 8:**
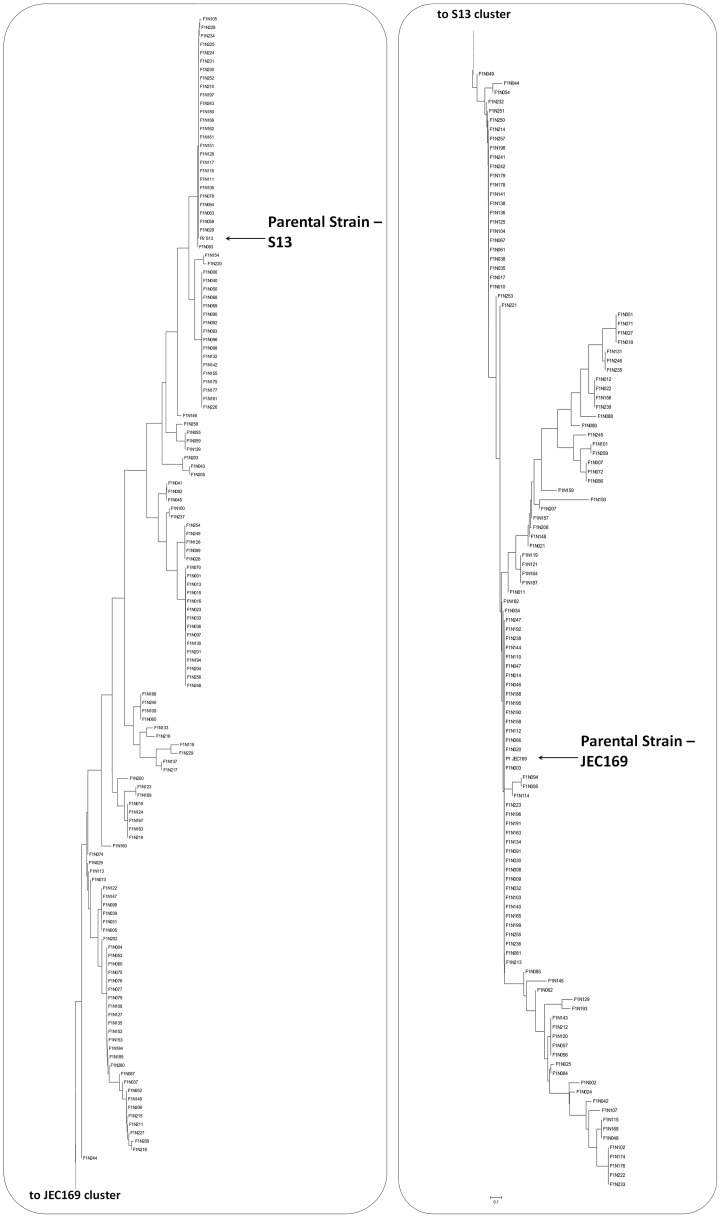
Genetic diversity among meiotic progeny from the laboratory cross between strains JEC169 and S13. The tree was constructed using the Neighbor-Joining method based on the PCR-RFLP data listed in Table 3 and Table S2. The two parental strains are highlighted by the arrows.

The majority of the F1 progeny (>90%) had 0 to 2 crossing-overs within this region (Table 3, Table 4, and Table S2), and there is extensive genetic diversity among the F1 progeny (Figure 8). The order of the markers in the linkage map is consistent with that in the physical map (Figure 7B). Our results showed that the recombination frequencies (calculated as “kb/(recombination event/100 progeny)”) between adjacent marker pairs ranged between 2.07 and 6.44, and the average recombination frequency within the chromosomal region covered by these markers (calculated as [Total Physical Distance]/[Total Genetic Distance]) was 3.11. Of the seven chromosomal regions, six of them had a recombination frequency that was considerably lower than the frequency of the gene conversion at the GC peak region (estimated to be between 0.78 and 2.72) (Figure 7). Not surprisingly, the only region that showed possibly higher recombination frequency than the gene conversion frequency was between the *CND05310* and *SXI1*/*SXI2* genes that encompass the region flanking the *MAT* locus, which was previously shown to exhibit an elevated recombination frequency during meiosis [13]. Although it is not possible to perform statistical analyses given the small number of gene conversion events observed, these results provide evidence that the frequency of the gene conversion at the GC peak region was at least comparable to, and likely greater than the average recombination frequency in other regions on the same chromosome during meiosis.

**Table 4:** Number of cross-overs among F1 progeny on chromosome 4.^a^

# of cross-overs^b^	0	1	2	3	4	Total
Including the *SXI1/2* gene	66	102	65	19	3	296
Excluding the *SXI1/2* gene	106	91	51	6	1	213

a : The chromosome 4 region covered by the PCR-RFLP markers.

b : Inferred from the PCR-RFLP genotyping data (Table 3 and Table S2).

## Discussion

Meiotic recombination is thought to be repressed over the majority of the heterogametic sex chromosomes (e.g. mammalian X and Y chromosomes), as well as the *MAT* loci in some fungi (e.g. the *MAT* locus in *C. neoformans*). Although this ensures the integrity of these highly rearranged chromosomal regions and maintains the divergence between alleles from opposite sexes (mating types), the lack of meiotic recombination also means these regions are effectively asexual, and thus under the threat of gradual deterioration due to 1) the accumulation of deleterious mutations that can no longer be eliminated efficiently through recombination, and 2) the irreversible manner in which the deleterious mutations accumulate within these regions (i.e. Muller's ratchet effect) [36], [37].

In the current study, we showed that within the *MAT* locus there is a GC rich intergenic region between the *RPO41* and *BSP2* genes that exists in all of the lineages of *C. neoformans* and *C. gattii* (Figure 1). The nucleotide composition of this GC rich region is diverged among different lineages while relatively conserved within each lineage. These observed population genetics patterns reflect the descent from a common ancestor of this GC rich intergenic region in all of the *C. neoformans* and *C. gattii* lineages, and the observed divergence among these lineages is the result of independent accumulation of mutations within this intergenic region in different lineages after their split from a common ancestor.

Recombination is thought to be repressed within the *MAT* locus of *C. neoformans*, due to sequence divergence and chromosomal rearrangements existing within the *MAT* locus between the two opposite mating types. If this is the case, the phylogenies of the three sections (the two flanking genes and the intergenic region) should have consistent topologies. In addition, the alleles from opposite mating types should exhibit independent accumulation of mutations, and thus their phylogeny should have a mating type specific topology; that is, alleles cluster together based on the mating types of the strains in which they reside. Furthermore, because no signal of positive selection was detected in the two flanking genic regions, the intergenic region should have accumulated more polymorphisms than regions corresponding to the two flanking genes, in which possible mechanisms such as purifying selection could slow down the accumulation of mutations and maintain sequence identity. However, this is in contrast to the results from our analyses. Specifically, the topologies of the two flanking genes were statistically different from those of the intergenic region when serotype A or serotype D alleles were analyzed. Additionally, among the serotype D alleles, the intergenic GC rich region showed a comparable level of polymorphism when compared to the two flanking genic regions (*RPO41* and *BSP2*), and a lower level of polymorphism when compared to other genes and intergenic regions that are also located within the *MAT* locus (Table 2). Furthermore, we found evidence of allele sharing between strains of opposite mating types in both the serotype A and D populations (Figure 4, Figure 5), as well as gene conversion tracts around the GC rich intergenic region among serotype A strains (Figure 6). Thus, our results instead support a model in which among serotype A and D *C. neoformans* strains, respectively, there is still ongoing gene flow between the two mating types at the GC rich intergenic region within the *MAT* locus.

This hypothesis is supported by observations from the laboratory sexual cross. By collecting and analyzing a large number of meiotic progeny from a laboratory cross, we found that recombination, in the form of gene conversion, occurred around the GC rich intergenic region during sexual reproduction in serotype D *C. neoformans*. Interestingly, in both of the gene conversion events identified among meiotic progeny, the direction of the gene conversion was A→G, consistent with previous studies indicating that gene conversion at recombination hot spots shows a bias favoring G/C over A/T [25], [27], [38]. However, it could also be that this uni-directional gene conversion is actually favoring *MAT* **a** alleles over *MAT*α alleles. This could be investigated by analyzing meiotic progeny of a laboratory cross, in which the *MAT* **a** parent has A or T, while the *MAT*α parent has G or C alleles at the polymorphic sites within the intergenic GC rich region.

The frequency of this gene conversion event was estimated to be between 0.78 and 2.72 kb/(events/100 progeny). This is at least comparable to the meiotic recombination frequencies observed in other regions of chromosome 4 (Figure 7). The genome wide meiotic recombination of serotype D *C. neoformans* has been previously estimated to be ∼13.2 kb/cM [39], which is considerably lower than the meiotic recombination frequency that we observed at the other regions of chromosome 4 (ranged between 2.07 and 6.44 kb/cM). However, in the study by Marra et al. [39], several of the chromosomes were each composed of multiple linkage groups, suggesting an underestimation of the genetic distances across the genome (those recombination events between the linkage groups), and thus an overestimation of the genomic average recombination frequency. Unfortunately, chromosome 4 was one of the chromosomes that were composed of two separate linkage groups, preventing us from comparing directly the results from the two studies. Another possibility is that chromosome 4 could experience a higher than average recombination frequency during meiosis. It has been shown that in *C. neoformans* during serotype A and D hybridization, although most of the chromosomes experience significantly reduced recombination frequency, the regions on chromosome 4 still showed levels of recombination that are comparable to those observed in intra-variety mating [40]. It is possible that there is an intrinsic mechanism that promotes recombination along chromosome 4 during sexual reproduction, which could result from the *MAT* locus residing on chromosome 4. This could be investigated by genome wide analysis of the meiotic recombination frequencies at markers from different chromosomes and with different GC content profiles. Taken together, our analyses support the conclusion that gene conversion is occurring around the GC rich intergenic region at a frequency that is at least comparable to, and likely higher than the typical meiotic recombination frequencies in other genomic regions during sexual reproduction of *C. neoformans*.

**Figure 9:**
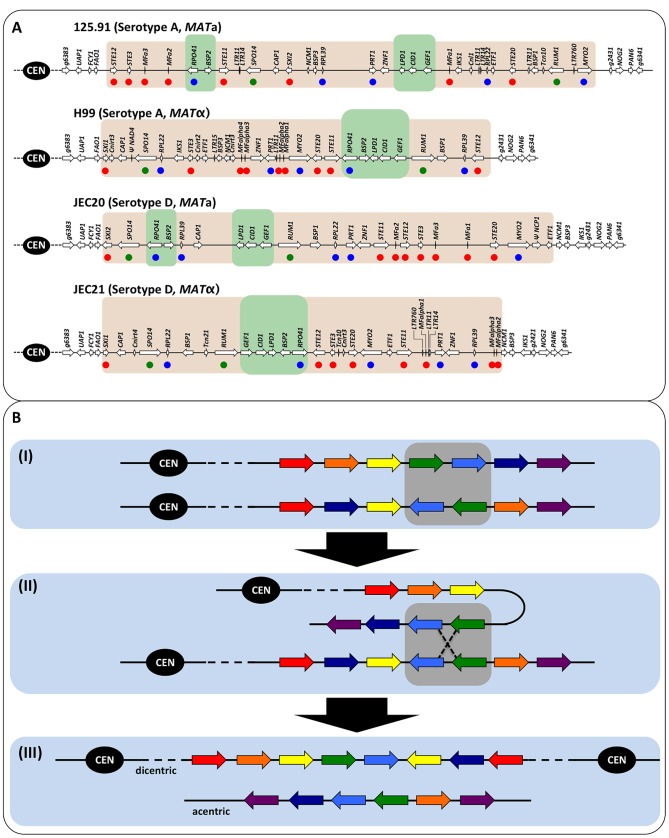
Diagrams of the *MAT* loci of *C. neoformans* var. *grubii* and var. *neoformans* and illustration of meiotic recombination within inverted chromosomal region. (A) Regions shaded in gray indicate the *MAT* loci. Green shading indicates the gene clusters, within which the *RPO41* and *BSP2* genes are located, and which have been hypothesized to be the most recent strata that was recruited into the *MAT* locus. Red dots denote the genes involved in mating and/or meiosis, green dots indicate hypothetical meiotic genes, and blue dots highlight known essential genes. (B) The black circle labeled “CEN” is the centromere. The seven arrows with different colors indicate seven different genes. The balanced composition of each chromosome at these seven genes is indicated by the presence of one gene of each of the seven colors (e.g., the two chromosomes in (I)). Gray shading highlights the two genes, green and blue, that are inverted. (I) The two chromatids involved in meiotic recombination are shown. (II) Inverted chromosomal regions align during meiosis, and meiotic recombination occurs within the inverted region. (III) Meiotic recombination within the inverted chromosomal region results in two chromosomes that are imbalanced in their gene compositions, and are acentric and dicentric, respectively.

It is not yet clear how gene conversion occurs at this intergenic GC rich region. The two flanking genes, *RPO41* and *BSP2*, are among a group of genes that constitutes a syntenic cluster with the same orientation between *MAT* **a** and *MAT*α alleles in serotype A, whereas this gene pair is oppositely oriented between *MAT* **a** and *MAT*α alleles in serotype D (Figure 9). A previous study has hypothesized that this gene cluster represents a strata that was recruited into the *MAT* locus most recently during evolution [41]. Thus, it could be that the repression of recombination is less severe, and recombination can still be initiated within this region. It has been shown that recombination hot spots flank the *MAT* locus of *C. neoformans*, and these hot spots are associated with chromosomal regions with a high GC content [13]. Thus, it is possible that the factors responsible for initiating a high frequency recombination at the flanking regions of *MAT* locus could also recognize the GC rich intergenic region between the *RPO41* and *BSP2* genes within the *MAT* locus, and induce lesions such as double-strand breaks (DSBs) that promote recombination within this region. Additionally, DSBs could also be induced in this region because of the high susceptibility of this region to mutagenic factors due to their high GC content [28]. The DSB could then be repaired through either crossing-over or gene conversion. However, due to the rearranged chromosomal locations within the *MAT* loci, as well as the opposite orientations (in serotype D) of the *MAT* **a** and *MAT*α alleles, typical crossing-over within the GC rich intergenic region would result in abnormal chromosomes, such as dicentric or acentric chromosomes, and/or chromosomes with duplications and deletions of a variety of essential genes or genes involved in mating and meiosis (Figure 9). As a consequence, the progeny inheriting these abnormal chromosomes would likely be inviable. On the other hand, DSBs repaired through gene conversion do not produce abnormal chromosomes, thus resulting in the bias that only gene conversion events can be recovered from the meiotic recombination events that had actually occurred at this GC rich intergenic region during sexual reproduction. If this is the case, our estimation of the recombination frequency at this GC rich intergenic region would be an underestimation of the actual recombination frequency (including both nonviable cross-overs and viable gene conversions) within this region.

It is also possible that recombination, in the form of gene conversion, could actually be initiated and carried out actively during meiosis within the GC rich intergenic region, and maybe even in other regions of the *MAT* locus of *C. neoformans*. One of the best studied examples of active initiation of gene conversion during mitosis is mating type switching in the yeast *S. cerevisiae* through gene conversion initiated by the HO endonuclease, which enables haploid *Saccharomyces* cells to have the potential to change mating type as often as every mitotic generation [42], [43]. Active initiation of gene conversion within the *C. neoformans MAT* locus could have two consequences. First, it might help align the opposite mating type alleles during meiosis. The *MAT* locus of *C. neoformans* is unusually large (>100 kb), and is highly rearranged between alleles of opposite mating type. Although it has been widely accepted that meiosis in almost all species requires at least one crossover event per chromosome, the highly diverged and rearranged nature between alleles of opposite mating types could still pose a problem for the proper alignment between opposite alleles during meiosis. Thus, a recombination event within the *MAT* locus could help in generating the tension required between homologous chromosomes for their proper segregation during meiosis I. Second, full repression of recombination within the *MAT* locus of *C. neoformans* would render this region asexual, and thus subject the *MAT* locus to gradual deterioration due to the irreversible accumulation of mutations within the *MAT* (i.e. Muller's ratchet [36]). It has been shown that five of the 20 genes within the *MAT* locus are essential (including the *RPO41* gene) [41], and although mechanisms such as purifying selection could act to prevent mutations from accumulating within these genes, the maintenance of sequence identity of these essential genes could nonetheless be facilitated by gene conversion events occurring in these regions. This is similar to situations where gene conversion that is biased against new mutations has been proposed to slow the observed mutation rate in plants and bacteria [44], [45]. This is also consistent with recent simulation-based studies that suggest that even low levels of gene conversion are sufficient to maintain the sequence integrity of the genes located on the human Y chromosome [46], [47]. Alternatively, this GC rich intergenic region may represent an ancient recombination hot spot that was adjacent to the essential gene, *RPO41*, before it was recruited into the *MAT* locus, as demonstrated by a study showing that purging of deleterious mutations in essential genes may be an important factor driving meiotic crossover [48].

Recombination, including gene conversion, has been reported to occur at chromosomal regions within which recombination has been thought to be repressed, such as centromeric regions [15]. Recent studies provide further empirical evidence that recombination is more universal, and is occurring at considerably higher frequencies within these “non-recombining” regions. It has been shown that in mouse crossing-over within the PAR region located on the otherwise non-recombining sex chromosomes is critical for male meiosis [29], [30]. Additionally, gene conversion has been discovered to be widespread within the centromeric regions of maize [14]. Furthermore, occasional X-Y recombination, as well as gene conversion, has been found to be the most plausible explanation for the observed sex-chromosome homomorphy in European tree frogs [49]. These recombination events play important roles in shaping the evolutionary trajectories of the chromosomal regions that are involved. A recent study by Sloan et al proposed that changes in recombination frequency (including gene conversion) are a central force driving the evolution of mitochondrial genome structure [50]. It is possible that many recombination events occurring in these supposed “non-recombining” regions might have gone unnoticed, partly due to the fact that in many cases, recombination might have taken the form of gene conversion, which can be detected only when sequences involved are non-identical, and thus is less likely to be recorded than crossing-over. Our study provides further evidence that, indeed, some presumed recombination “cold spots” are actually not that cold, after all.

## Materials and Methods

### Strains and media

The strains used in this study are listed in Table 1, and their VN types have been determined in previous studies [51], [52], [53] Strain JEC169 is an *ade2 ura5* auxotrophic isolate derived from JEC20 [54]. The *ADE2* and *URA5* genes are located on chromosomes 5 and 7, respectively. All of the other strains are clinical or environmental isolates. All of the strains were grown and maintained on YPD agar plates unless mentioned otherwise.

### Laboratory cross and screening of recombinant progeny

A cross between strains JEC169 (*MAT* **a** *ade2 ura5*) and S13 (*MAT*α *ADE2 URA5*) was conducted on V8 agar medium plates as previously described [55]. After 2 weeks of incubation at room temperature in the dark, the plate was inspected by light microscopy. The area outside the edge of the mating mixture containing abundant basidiospores was excised, and suspended in water. The suspension was then diluted and spread onto YPD agar plates. After two days of incubation at 30°C, the colonies that appeared on the YPD plates were transferred onto testing plates, SD-ade and SD-ura [56], to confirm their genotypes at the auxotrophic markers. The colonies that only grew on one of the two testing plates were considered to be recombinant, and were then stored in 35% glycerol at −80°C for the following studies.

### Mating assays

The mating type of each recombinant progeny was determined by backcrossing the progeny with the two parental strains. The mating assay was performed in the same way as the laboratory cross described above. Mating was determined to be successful if hyphae, basidia, and basidiospore chains were observed after 2 weeks of incubation.

### DNA isolation, PCR amplification, enzyme digestion, and sequencing reactions

DNA isolation and PCR amplification were carried out as previously described [57]. Primers used in this study are listed in Table S1. For serotype AD strains, different primer pairs were used to amplify the serotype A and serotype D specific alleles of the *RPO41*-*BSP2* region, respectively (Table S1). All of the PCR reactions were carried out using an annealing temperature of 60°C. Restriction enzyme digestions were performed according to the manufacturer's instructions (New England Laboratory Casework Co., Inc.). Sequencing reactions were conducted at the Genome Sequencing & Analysis Core Facility at the Duke Institute for Genome Sciences & Policy.

### Population genetic analyses

Sequence alignments were carried out using ClustalX 2.1 [58]. The aligned sequences were then curated manually using the software MacClade 4.06 [59]. MEGA 5 [60] was used for the phylogeny constructions and population genetics analyses. Bootstrap support for the phylogenetic trees was determined by performing 1000 bootstraps. Kishino-Hasegawa test of topologies was carried using the SHTest implemented in the software PAUP 4.0 [61]. Gene conversion events were detected using program GENECONV v.1.81 following the software's instruction [62]. Significant gene conversion tracts were identified if the Global-P value was smaller than 0.05 based on permutation.

### Linkage map construction

A linkage map was constructed using the software program MapMaker [63]. The chromosomal location of each marker was estimated as the position of the midpoint of the PCR product of that marker in the sequence of strain JEC21 chromosome 4 (GenBank: AE017344).

## Supporting Information

Table S1 Primers used in this study.
(DOCX)

Table S2 Genotypes of the two parental strains and 255 recombinant progeny at the 18 markers screened in this study.
(DOCX)
