# Supplementary material for: Gene Conversion Occurs within the Mating-Type Locus of Cryptococcus neoformans during Sexual Reproduction
Source: PLoS Genet. 2012 Jul 5;8(7):e1002810. doi: 10.1371/journal.pgen.1002810 (PMC3390403; doi:10.1371/journal.pgen.1002810)
Supplement: Table S1 — Primers used in this study. (DOCX) [file pgen.1002810.s001.docx]

Table S1. Primers used in this study

| **Primer** | **Sequence** | **Tm in PCR** | **Purpose** | **Restriction Enzyme** |
| --- | --- | --- | --- | --- |
| JOHE17985 | CACGTTCTTCTTGATCGTCAG | 60 | PCR amplification of *RPO41-BSP2* region | n.a. |
| JOHE17988 | AGGACTCTTTATAGCGCATA | 60 |  |  |
| JOHE26408 | ATTGGGTGAACGTAAAGCAGC | 60 | PCR amplification of serotype A specific allele of *RPO41-BSP2* region | n.a. |
| JOHE26409 | GCGCTCCTTTGTCGCTTAGC | 60 |  |  |
| JOHE26410 | ATTGGGCGAGCGTAAAGCGGA | 60 | PCR amplification of serotype D specific allele of *RPO41-BSP2* region | n.a. |
| JOHE26411 | CCGCTCCCTTGTCGCTTAGT | 60 |  |  |
| JOHE22850 | GCGAACCGAGCTTCATTACG | n.a. | Sequencing of the *RPO41*-*BSP2* PCR products | n.a. |
| JOHE22980 | GGGTGAGGTTGCCTACAGTTGCGA | n.a. |  |  |
| JOHE26478 | ATACCATCAGCTATACCGCC | n.a. |  |  |
| JOHE26479 | TGGCGTCGTTTCTCCAGTAC | n.a. |  |  |
| JOHE23355 | TGCGATCTTGGCGAGTTCGTC | 60 | PCR amplification of fragment from the *CND03670* gene [PCR-RFLP marker] | HaeIII + MspI |
| JOHE23356 | CGTTGGAATCCAGCGGGAAA | 60 |  |  |
| JOHE23357 | TCCAGTTCCGCTGGCGTTTTG | 60 | PCR amplification of fragment from the *CND03960* gene [PCR-RFLP marker] | HindIII |
| JOHE23358 | TCCGGGCAACTTTACCGCAA | 60 |  |  |
| JOHE23359 | TTCCTGAACCGCTTGGCGAAAA | 60 | PCR amplification of fragment from the *CND04120* gene [PCR-RFLP marker] | HaeIII |
| JOHE23360 | ATCAAATTGCCCCCGGCGAT | 60 |  |  |
| JOHE23361 | TTCGTTCAAGCAGCCGCCATAC | 60 | PCR amplification of fragment from the *CND04340* gene [PCR-RFLP marker] | HaeIII |
| JOHE23362 | AAACTCCCGGCATTGCCGATG | 60 |  |  |
| JOHE23363 | ACAAATCTGGTTCGGGGCCT | 60 | PCR amplification of fragment from the *CND04540* gene [PCR-RFLP marker] | HphI |
| JOHE23364 | TCAGCGCGTTTTACGCCAAG | 60 |  |  |
| JOHE23367 | TCCCATCCGGCATGGAATGA | 60 | PCR amplification of fragment from the *CND05140* gene [PCR-RFLP marker] | HinfI |
| JOHE23368 | TTGACTTCAACGCCGCTCGACTT | 60 |  |  |
| JOHE23369 | TGCCCGCGTTCAAAAGCTCAC | 60 | PCR amplification of fragment from the *CND05310* gene [PCR-RFLP marker] | AccI + PvuII |
| JOHE23370 | ATGGACGTTCCCAGTTGCGAT | 60 |  |  |
| JOHE17409 | GCCGTGCAAGGGTGTAGG | 60 | PCR amplification of fragment from the *SXI1* gene | n.a. |
| JOHE14895 | GGGCCATTGGAGGAAGCTG | 60 |  |  |
| JOHE21653 | ATGGGCAGCAACCTTGACATC | 60 | PCR amplification of fragment from the *SXI2* gene | n.a. |
| JOHE21654 | GGATAGATCTTACCCCCTGAGGACTGT | 60 |  |  |
