# Supplementary material for: Gene Conversion Occurs within the Mating-Type Locus of Cryptococcus neoformans during Sexual Reproduction
Source: PLoS Genet. 2012 Jul 5;8(7):e1002810. doi: 10.1371/journal.pgen.1002810 (PMC3390403; doi:10.1371/journal.pgen.1002810)
Supplement: Table S2 — Genotypes of the two parental strains and 255 recombinant progeny at the 18 markers screened in this study. (DOCX) [file pgen.1002810.s002.docx]

Table S2. Genotypes of the two parental strains and 255 recombinant progeny at the 18 markers screened in this study

| Isolate | Phenotypic Markers | | | Polymorphic sites across *RPO41* and *BSP2* genes | | | | | | | | PCR-RFLP markers located at other genes on chromosome 4 ^e^ | | | | | | | | Number of crossing-overs between the *CND03670* gene and the *SXI1/SXI2* gene |
| --- | --- | --- | --- | --- | --- | --- | --- | --- | --- | --- | --- | --- | --- | --- | --- | --- | --- | --- | --- | --- |
|  | Mating Type ^a^ | ADE ^b^ | URA ^b^ | 2317 ^c^ | 2374 ^c^ | 2548 ^c^ | 2960 ^c^ | 2966 ^c^ | 4023 ^d^ | 4413 ^d^ | 5100 ^e^ | CND03670 ^f^ | CND03960 ^f^ | CND04120 ^f^ | CND04340 ^f^ | CND04540 ^f^ | CND05140 ^f^ | CND05310 ^f^ | SXI1/SXI2 ^g^ |  |
| JEC169 | **a** | - | - | T | G | T | G | G | G | G | G | a | a | a | a | a | a | a | a | n.a. |
| S13 | α | + | + | A | A | C | A | A | A | A | A | b | b | b | b | b | b | b | b | n.a. |
| F1N001 ^h^ | α | + | - | A | A | C | A | A | A | A | A | a | b | b | b | b | b | b | b | 1 |
| F1N002 | n.a. | + | - | T | G | T | G | G | G | G | G | a | a | b | b | b | a | a | a | 2 |
| F1N003 | **a** | + | - | T | G | T | G | G | G | G | G | a | a | a | a | a | a | a | a | 0 |
| F1N004 | α | + | - | A | A | C | A | A | A | A | A | a | a | a | a | a | b | b | b | 1 |
| F1N005 | n.a. | + | - | T | G | T | G | G | G | G | G | a | a | a | a | a | b | b | a | 2 |
| F1N006 | **a** | + | - | T | G | T | G | G | G | G | G | b | b | b | b | b | b | b | a | 1 |
| F1N007 | **a** | + | - | A | A | C | A | A | A | A | A | b | b | a | a | a | b | b | b | 2 |
| F1N008 | **a** | + | - | T | G | T | G | G | G | G | G | a | a | a | a | a | a | a | a | 0 |
| F1N009 | **a** | + | - | T | G | T | G | G | G | G | G | a | a | a | a | a | a | a | a | 0 |
| F1N010 | α | + | - | A | A | C | A | A | A | A | A | a | a | a | a | a | a | a | b | 1 |
| F1N011 | n.a. | + | - | T | G | T | G | G | G | G | G | a | a | a | a | a | a | a | a | 0 |
| F1N012 | α | + | - | A | A | C | A | A | A | A | A | b | b | b | a | a | a | a | b | 2 |
| F1N013 | α | + | - | A | A | C | A | A | A | A | A | a | b | b | b | b | b | b | b | 1 |
| F1N014 | **a** | + | - | T | G | T | G | G | G | G | G | a | a | a | a | a | a | a | a | 0 |
| F1N015 | α | + | - | A | A | C | A | A | A | A | A | a | b | b | b | b | b | b | b | 1 |
| F1N016 | α | + | - | A | A | C | A | A | A | A | A | a | b | b | b | b | b | b | b | 1 |
| F1N017 | α | + | - | A | A | C | A | A | A | A | A | a | a | a | a | a | a | a | b | 1 |
| F1N018 | α | + | - | T | G | T | G | G | G | G | G | b | b | b | b | a | a | a | a | 1 |
| F1N019 | α | + | - | A | A | C | A | A | A | A | A | a | a | a | a | b | b | b | b | 1 |
| F1N020 | **a** | + | - | T | G | T | G | G | G | G | G | a | a | a | a | a | a | a | a | 0 |
| F1N021 | α | + | - | A | A | C | A | A | A | A | A | b | a | a | a | a | a | a | b | 2 |
| F1N022 | α | + | - | A | A | C | A | A | A | A | A | b | b | b | a | a | a | a | b | 2 |
| F1N023 | α | + | - | A | A | C | A | A | A | A | A | a | b | b | b | b | b | b | b | 1 |
| F1N024 | α | + | - | A | A | C | A | A | A | A | A | a | a | b | b | b | a | a | b | 3 |
| F1N025 | α | + | - | A | A | C | A | A | A | A | A | a | a | a | b | b | a | a | b | 3 |
| F1N026 | **a** | + | - | T | G | T | G | G | G | G | G | a | b | b | b | b | b | b | a | 2 |
| F1N027 | **a** | + | - | T | G | T | G | G | G | G | G | b | b | b | b | a | a | a | a | 1 |
| F1N028 | α | + | - | A | A | C | A | A | A | A | A | b | b | b | b | b | b | b | b | 0 |
| F1N029 | α | + | - | A | A | C | A | A | A | A | A | a | a | a | a | a | b | b | b | 1 |
| F1N030 | **a** | + | - | T | G | T | G | G | G | G | G | a | a | a | a | a | a | a | a | 0 |
| F1N031 | n.a. | + | - | T | G | T | G | G | G | G | G | a | a | a | a | a | b | b | a | 2 |
| F1N032 | **a** | + | - | T | G | T | G | G | G | G | G | a | a | a | a | a | a | a | a | 0 |
| F1N033 | α | + | - | A | A | C | A | A | A | A | A | a | b | b | b | b | b | b | b | 1 |
| F1N034 | **a** | + | - | T | G | T | G | G | G | G | G | a | a | a | a | a | a | a | a | 0 |
| F1N035 | α | + | - | A | A | C | A | A | A | A | A | a | a | a | a | a | a | a | b | 1 |
| F1N036 | α | + | - | A | A | C | A | A | A | A | A | a | b | b | b | b | b | b | b | 1 |
| F1N037 | α | + | - | A | A | C | A | A | A | A | A | b | a | a | a | a | b | b | b | 2 |
| F1N038 | α | + | - | A | A | C | A | A | A | A | A | a | a | a | a | a | a | a | b | 1 |
| F1N039 | n.a. | + | - | T | G | T | G | G | G | G | G | a | a | a | a | a | b | b | a | 2 |
| F1N040 | **a** | + | - | T | G | T | G | G | G | G | G | b | b | b | b | b | b | b | a | 1 |
| F1N041 | α | + | - | A | A | C | A | A | A | A | A | a | b | b | b | a | b | b | b | 3 |
| F1N042 | α | + | - | A | A | C | A | A | A | A | A | a | b | b | b | b | a | a | b | 3 |
| F1N043 | **a** | + | - | T | G | T | G | G | G | G | G | b | b | b | b | b | b | a | a | 1 |
| F1N044 | α | + | - | A | A | C | A | A | G | G | A | a | a | a | a | a | a | b | b | 1 |
| F1N045 | α | + | - | A | A | C | A | A | A | A | A | a | b | b | b | a | b | b | b | 3 |
| F1N046 | n.a. | + | - | T | G | T | G | G | G | G | G | a | a | a | a | a | a | a | a | 0 |
| F1N047 | **a** | + | - | T | G | T | G | G | G | G | G | a | a | a | a | a | a | a | a | 0 |
| F1N048 | α | + | - | A | A | C | A | A | A | A | A | b | b | b | b | b | a | a | b | 2 |
| F1N049 | α | + | - | A | A | C | A | A | A | A | A | a | a | a | a | a | a | a | b | 1 |
| F1N050 | **a** | + | - | T | G | T | G | G | G | G | G | b | b | b | b | b | b | b | a | 1 |
| F1N051 | **a** | + | - | T | G | T | G | G | G | G | G | b | b | b | b | a | a | a | a | 1 |
| F1N052 | α | + | - | A | A | C | A | A | A | A | A | b | a | a | a | a | b | b | b | 2 |
| F1N053 | α | + | - | A | A | C | A | A | A | A | A | a | a | a | a | a | b | b | b | 1 |
| F1N054 | α | + | - | A | A | C | A | A | A | A | A | a | a | a | a | a | a | a | b | 1 |
| F1N055 | α | + | - | A | A | C | A | A | A | A | A | b | b | b | b | a | b | b | b | 2 |
| F1N056 | n.a. | + | - | T | G | T | G | G | G | G | G | a | a | a | b | b | a | a | a | 2 |
| F1N057 | **a** | + | - | T | G | T | G | G | G | G | G | a | a | a | b | b | a | a | a | 2 |
| F1N058 | α | + | - | A | A | C | A | A | A | A | A | b | b | b | b | b | b | b | b | 0 |
| F1N059 | α | + | - | A | A | C | A | A | A | A | A | b | b | b | b | a | b | b | b | 2 |
| F1N060 | α | + | - | A | A | C | A | A | A | A | A | a | a | a | a | a | b | b | b | 1 |
| F1N061 | α | + | - | A | A | C | A | A | A | A | A | a | a | a | a | a | a | a | b | 1 |
| F1N062 | **a** | + | - | T | G | T | G | G | G | G | G | a | a | a | a | b | a | a | a | 2 |
| F1N063 | α | + | - | A | A | C | A | A | A | A | A | b | b | b | b | b | b | b | b | 0 |
| F1N064 | α | + | - | A | A | C | A | A | A | A | A | b | b | b | b | b | b | b | b | 0 |
| F1N065 | α | + | - | A | A | C | A | A | A | A | A | a | a | a | b | b | b | b | b | 1 |
| F1N066 | **a** | + | - | T | G | T | G | G | G | G | G | a | a | a | a | a | a | a | a | 0 |
| F1N067 | α | + | - | A | A | C | A | A | A | A | A | a | a | a | a | a | a | a | b | 1 |
| F1N068 | **a** | + | - | T | G | T | G | G | G | G | G | b | b | b | b | b | b | b | a | 1 |
| F1N069 | **a** | + | - | T | G | T | G | G | G | G | G | b | b | b | b | b | b | b | a | 1 |
| F1N070 | α | + | - | A | A | C | A | A | A | A | A | a | b | b | b | b | b | b | b | 1 |
| F1N071 | **a** | + | - | T | G | T | G | G | G | G | G | b | b | b | b | a | a | a | a | 1 |
| F1N072 | α | - | + | A | A | C | A | A | A | A | A | b | b | a | a | a | b | b | b | 2 |
| F1N073 | α | + | - | A | A | C | A | A | A | A | A | a | a | a | a | a | b | b | b | 1 |
| F1N074 | α | + | - | A | A | C | A | A | A | A | A | a | a | a | a | a | b | b | b | 1 |
| F1N075 | α | + | - | A | A | C | A | A | A | A | A | a | a | a | a | a | b | b | b | 1 |
| F1N076 | α | + | - | A | A | C | A | A | A | A | A | a | a | a | a | a | b | b | b | 1 |
| F1N077 | α | + | - | A | A | C | A | A | A | A | A | a | a | a | a | a | b | b | b | 1 |
| F1N078 | α | + | - | A | A | C | A | A | A | A | A | b | b | b | b | b | b | b | b | 0 |
| F1N079 | n.a. | + | - | A | A | C | A | A | A | A | A | a | a | a | a | a | b | b | b | 1 |
| F1N080 | **a** | + | - | T | G | T | G | G | G | G | G | b | b | b | a | a | a | a | a | 1 |
| F1N081 | **a** | + | - | T | G | T | G | G | G | G | G | a | a | a | a | a | a | a | a | 0 |
| F1N082 | α | + | - | A | A | C | A | A | A | A | A | a | b | b | b | a | b | b | b | 3 |
| F1N083 | α | + | - | A | A | C | A | A | A | A | A | b | b | b | b | b | b | b | b | 0 |
| F1N084 | n.a. | + | - | T | G | T | G | G | G | G | G | a | a | a | b | b | a | a | a | 2 |
| F1N085 | **a** | + | - | T | G | T | G | G | G | G | G | a | a | a | a | b | a | a | a | 2 |
| F1N086 | α | + | - | A | A | C | A | A | A | A | A | b | b | a | a | a | b | b | b | 2 |
| F1N087 | α | + | - | A | A | C | A | A | A | A | A | a | a | a | a | a | b | b | b | 1 |
| F1N088 | α | + | - | A | A | C | A | A | A | A | A | b | b | b | a | a | a | b | b | 2 |
| F1N089 | **a** | + | - | T | G | T | G | G | G | G | G | a | b | b | b | b | b | b | a | 2 |
| F1N090 | **a** | + | - | T | G | T | G | G | G | G | G | b | b | b | b | b | b | b | a | 1 |
| F1N091 | n.a. | + | - | T | G | T | G | G | G | G | G | a | a | a | a | a | a | a | a | 0 |
| F1N092 | **a** | + | - | T | G | T | G | G | G | G | G | b | b | b | b | b | b | b | a | 1 |
| F1N093 | **a** | + | - | T | G | T | G | G | G | G | G | b | b | b | b | b | b | b | a | 1 |
| F1N094 | **a** | + | - | T | G | T | G | G | G | G | G | a | a | a | a | a | b | a | a | 2 |
| F1N095 | **a** | + | - | T | G | T | G | G | G | G | G | a | a | a | a | a | b | a | a | 2 |
| F1N096 | **a** | + | - | T | G | T | G | G | G | G | G | b | b | b | b | b | b | b | a | 1 |
| F1N097 | α | + | - | A | A | C | A | A | A | A | A | a | b | b | b | b | b | b | b | 1 |
| F1N098 | **a** | + | - | T | G | T | G | G | G | G | G | b | b | b | b | b | b | b | a | 1 |
| F1N099 | **a** | + | - | T | G | T | G | G | G | G | G | a | a | a | a | a | b | b | a | 2 |
| F1N100 | α | + | - | A | A | C | A | A | A | A | A | a | b | b | b | b | b | a | b | 3 |
| F1N101 | **a** | + | - | T | G | T | G | G | G | G | G | b | b | a | a | a | b | b | a | 3 |
| F1N102 | **a** | + | - | T | G | T | G | G | G | G | G | b | b | b | b | b | a | a | a | 1 |
| F1N103 | **a** | + | - | T | G | T | G | G | G | G | G | a | a | a | a | a | a | a | a | 0 |
| F1N104 | n.a. | + | - | A | A | C | A | A | A | A | A | a | a | a | a | a | a | a | b | 1 |
| F1N105 | α | + | - | A | A | C | A | A | A | A | A | b | b | b | b | b | b | b | b | 0 |
| F1N106 | α | + | - | A | A | C | A | A | A | A | A | b | b | b | b | b | b | b | b | 0 |
| F1N107 | **a** | + | - | T | G | T | G | G | G | G | G | a | b | b | b | b | a | a | a | 2 |
| F1N108 | α | + | - | A | A | C | A | A | A | A | A | a | a | a | a | a | b | b | b | 1 |
| F1N109 | α | + | - | A | A | C | A | A | A | A | A | a | a | a | b | b | b | b | b | 1 |
| F1N110 | n.a. | + | - | T | G | T | G | G | G | G | G | a | a | a | a | a | a | a | a | 0 |
| F1N111 | α | + | - | A | A | C | A | A | A | A | A | b | b | b | b | b | b | b | b | 0 |
| F1N112 | n.a. | + | - | T | G | T | G | G | G | G | G | a | a | a | a | a | a | a | a | 0 |
| F1N113 | α | + | - | A | A | C | A | A | A | A | A | a | a | a | a | a | b | b | b | 1 |
| F1N114 | **a** | + | - | T | G | T | G | G | G | G | G | a | a | a | a | a | b | a | a | 2 |
| F1N115 | α | + | - | A | A | C | A | A | A | A | A | b | b | b | b | b | a | a | b | 2 |
| F1N116 | α | + | - | A | A | C | A | A | A | A | A | b | b | b | b | b | b | b | b | 0 |
| F1N117 | α | + | - | A | A | C | A | A | A | A | A | b | b | b | b | b | b | b | b | 0 |
| F1N118 | α | + | - | A | A | C | A | A | A | A | A | b | a | b | b | b | b | b | b | 2 |
| F1N119 | **a** | + | - | T | G | T | G | G | G | G | G | b | a | a | a | a | a | a | a | 1 |
| F1N120 | n.a. | + | - | T | G | T | G | G | G | G | G | a | a | a | b | b | a | a | a | 2 |
| F1N121 | **a** | + | - | T | G | T | G | G | G | G | G | b | a | a | a | a | a | a | a | 1 |
| F1N122 | **a** | + | - | T | G | T | G | G | G | G | G | a | a | a | a | a | b | b | a | 2 |
| F1N123 | α | + | - | A | A | C | A | A | A | A | A | b | a | a | a | b | b | b | b | 2 |
| F1N124 | α | + | - | A | A | C | A | A | A | A | A | a | a | a | a | b | b | b | b | 1 |
| F1N125 | α | + | - | A | A | C | A | A | A | A | A | a | a | a | a | a | a | a | b | 1 |
| F1N126 | **a** | + | - | T | G | T | G | G | G | G | G | a | b | b | b | b | b | b | a | 2 |
| F1N127 | α | + | - | A | A | C | A | A | A | A | A | a | a | a | a | a | b | b | b | 1 |
| F1N128 | α | + | - | A | A | C | A | A | A | A | A | b | b | b | b | b | b | b | b | 0 |
| F1N129 | α | + | - | A | A | C | A | A | A | A | A | b | a | a | b | b | a | a | b | 4 |
| F1N130 | α | + | - | A | A | C | A | A | A | A | A | a | b | b | b | b | b | b | b | 1 |
| F1N131 | α | + | - | A | A | C | A | A | A | A | A | b | b | b | b | a | a | a | b | 2 |
| F1N132 | **a** | + | - | T | G | T | G | G | G | G | G | b | b | b | b | b | b | b | a | 1 |
| F1N133 | α | + | - | A | A | C | A | A | A | A | A | b | a | a | b | b | b | b | b | 2 |
| F1N134 | **a** | + | - | T | G | T | G | G | G | G | G | a | a | a | a | a | a | a | a | 0 |
| F1N135 | α | + | - | A | A | C | A | A | A | A | A | a | a | a | a | a | b | b | b | 1 |
| F1N136 | n.a. | + | - | A | A | C | A | A | A | A | A | a | a | a | a | a | a | a | b | 1 |
| F1N137 | **a** | + | - | T | G | T | G | G | G | G | G | a | a | b | b | b | b | b | a | 2 |
| F1N138 | **a** | + | - | A | A | C | A | A | A | A | A | a | a | a | a | a | a | a | b | 1 |
| F1N139 | α | + | - | A | A | C | A | A | A | A | A | b | b | b | b | a | b | b | b | 2 |
| F1N140 | **a** | + | - | T | G | T | G | G | G | G | G | a | a | a | a | a | a | a | a | 0 |
| F1N141 | α | + | - | A | A | C | A | A | A | A | A | a | a | a | a | a | a | a | b | 1 |
| F1N142 | **a** | + | - | T | G | T | G | G | G | G | G | b | b | b | b | b | b | b | a | 1 |
| F1N143 | **a** | + | - | T | G | T | G | G | G | G | G | a | a | a | b | b | a | a | a | 2 |
| F1N144 | **a** | + | - | T | G | T | G | G | G | G | G | a | a | a | a | a | a | a | a | 0 |
| F1N145 | **a** | + | - | T | G | T | G | G | G | G | G | a | a | b | a | b | a | a | a | 4 |
| F1N146 | α | + | - | A | A | C | A | A | A | A | A | b | b | a | b | b | b | b | b | 2 |
| F1N147 | **a** | + | - | T | G | T | G | G | G | G | G | a | a | a | a | a | b | b | a | 2 |
| F1N148 | α | + | - | A | A | C | A | A | A | A | A | b | a | a | a | a | a | a | b | 2 |
| F1N149 | α | + | - | A | A | C | A | A | A | A | A | b | a | a | a | a | b | b | b | 2 |
| F1N150 | α | + | - | A | A | C | A | A | A | A | A | b | a | b | b | a | a | a | b | 4 |
| F1N151 | α | + | - | A | A | C | A | A | A | A | A | b | b | b | b | b | b | b | b | 0 |
| F1N152 | α | + | - | A | A | C | A | A | A | A | A | a | a | a | a | a | b | b | b | 1 |
| F1N153 | α | + | - | A | A | C | A | A | A | A | A | a | a | a | a | a | b | b | b | 1 |
| F1N154 | **a** | + | - | T | G | T | G | G | G | G | G | b | b | b | b | b | a | b | a | 3 |
| F1N155 | **a** | + | - | T | G | T | G | G | G | G | G | b | b | b | b | b | b | b | a | 1 |
| F1N156 | α | + | - | A | A | C | A | A | A | A | A | b | b | b | a | a | a | a | b | 2 |
| F1N157 | α | + | - | A | A | C | A | A | A | A | A | b | a | a | a | a | a | a | b | 2 |
| F1N158 | **a** | + | - | T | G | T | G | G | G | G | G | a | a | a | a | a | a | a | a | 0 |
| F1N159 | α | + | - | A | A | C | A | A | A | A | A | b | a | a | a | a | a | b | b | 2 |
| F1N160 | α | + | - | A | A | C | A | A | A | A | A | a | a | a | a | b | b | a | b | 3 |
| F1N161 | α | + | - | A | A | C | A | A | A | A | A | b | b | b | b | b | b | b | b | 0 |
| F1N162 | α | + | - | A | A | C | A | A | A | A | A | b | b | b | b | b | b | b | b | 0 |
| F1N163 | **a** | + | - | T | G | T | G | G | G | G | G | a | a | a | a | a | a | a | a | 0 |
| F1N164 | **a** | + | - | T | G | T | G | G | G | G | G | b | a | a | a | a | a | a | a | 1 |
| F1N165 | **a** | + | - | T | G | T | G | G | G | G | G | a | a | a | a | a | a | a | a | 0 |
| F1N166 | α | + | - | A | A | C | A | A | A | A | A | b | b | b | b | b | b | b | b | 0 |
| F1N167 | α | + | - | A | A | C | A | A | A | A | A | a | a | a | a | b | b | b | b | 1 |
| F1N168 | n.a. | + | - | A | A | C | A | A | A | A | A | b | b | b | b | b | a | a | b | 2 |
| F1N174 | **a** | + | - | T | G | T | G | G | G | G | G | b | b | b | b | b | a | a | a | 1 |
| F1N175 | **a** | + | - | T | G | T | G | G | G | G | G | b | b | b | b | b | b | b | a | 1 |
| F1N176 | **a** | + | - | T | G | T | G | G | G | G | G | b | b | b | b | b | a | a | a | 1 |
| F1N177 | **a** | + | - | T | G | T | G | G | G | G | G | b | b | b | b | b | b | b | a | 1 |
| F1N178 | n.a. | + | - | A | A | C | A | A | A | A | A | a | a | a | a | a | a | a | b | 1 |
| F1N179 | α | + | - | A | A | C | A | A | A | A | A | a | a | a | a | a | a | a | b | 1 |
| F1N180 | α | + | - | A | A | C | A | A | A | A | A | b | b | b | b | b | b | b | b | 0 |
| F1N181 | α | + | - | T | G | T | G | G | G | G | G | b | b | b | b | b | b | b | a | 1 |
| F1N182 | **a** | + | - | T | G | T | G | G | G | G | G | a | a | a | a | a | a | a | a | 0 |
| F1N183 | α | + | - | A | A | C | A | A | A | A | A | a | a | a | a | b | b | b | b | 1 |
| F1N184 | α | + | - | A | A | C | A | A | A | A | A | a | a | a | a | a | b | b | b | 1 |
| F1N185 | α | + | - | A | A | C | A | A | A | A | A | a | a | a | a | a | b | b | b | 1 |
| F1N186 | α | + | - | A | A | C | A | A | A | A | A | a | a | a | b | b | b | b | b | 1 |
| F1N187 | **a** | + | - | T | G | T | G | G | G | G | G | b | a | a | a | a | a | a | a | 1 |
| F1N188 | **a** | + | - | T | G | T | G | G | G | G | G | a | a | a | a | a | a | a | a | 0 |
| F1N189 | α | + | - | A | A | C | A | A | A | A | A | b | a | a | a | b | b | b | b | 2 |
| F1N190 | n.a. | + | - | T | G | T | G | G | G | G | G | a | a | a | a | a | a | a | a | 0 |
| F1N191 | **a** | + | - | T | G | T | G | G | G | G | G | a | a | a | a | a | a | a | a | 0 |
| F1N192 | **a** | + | - | T | G | T | G | G | G | G | G | a | a | a | a | a | a | a | a | 0 |
| F1N193 | **a** | + | - | T | G | T | G | G | G | G | G | b | a | a | b | b | a | a | a | 3 |
| F1N194 | α | + | - | A | A | C | A | A | A | A | A | a | b | b | b | b | b | b | b | 1 |
| F1N195 | n.a. | + | - | T | G | T | G | G | G | G | G | a | a | a | a | a | a | a | a | 0 |
| F1N196 | **a** | + | - | T | G | T | G | G | G | G | G | a | a | a | a | a | a | a | a | 0 |
| F1N197 | α | + | - | A | A | C | A | A | A | A | A | b | b | b | b | b | b | b | b | 0 |
| F1N198 | α | + | - | A | A | C | A | A | A | A | A | a | a | a | a | a | a | a | b | 1 |
| F1N199 | n.a. | + | - | T | G | T | G | G | G | G | G | a | a | a | a | a | a | a | a | 0 |
| F1N200 | **a** | + | - | T | G | T | G | G | G | G | G | a | a | a | a | b | b | b | a | 2 |
| F1N201 | α | + | - | A | A | C | A | A | A | A | A | a | b | b | b | b | b | b | b | 1 |
| F1N202 | **a** | + | - | T | G | T | G | G | G | G | G | a | a | a | a | a | b | b | a | 2 |
| F1N203 | **a** | + | - | T | G | T | G | G | G | G | G | b | b | b | b | b | b | a | a | 1 |
| F1N204 | α | + | - | A | A | C | A | A | A | A | A | a | b | b | b | b | b | b | b | 1 |
| F1N205 | **a** | + | - | T | G | T | G | G | G | G | G | b | b | b | b | b | b | a | a | 1 |
| F1N206 | n.a. | + | - | A | A | C | A | A | A | A | A | b | a | a | a | a | b | b | b | 2 |
| F1N207 | α | + | - | A | A | C | A | A | A | A | A | b | a | a | a | a | a | a | b | 2 |
| F1N208 | α | + | - | A | A | C | A | A | A | A | A | b | a | a | a | a | a | a | b | 2 |
| F1N209 | α | + | - | A | A | C | A | A | A | A | A | b | a | a | a | a | b | b | b | 2 |
| F1N210 | α | + | - | A | A | C | A | A | A | A | A | b | b | b | b | b | b | b | b | 0 |
| F1N211 | α | + | - | A | A | C | A | A | A | A | A | b | a | a | a | a | b | b | b | 2 |
| F1N212 | **a** | + | - | T | G | T | G | G | G | G | G | a | a | a | b | b | a | a | a | 2 |
| F1N213 | **a** | + | - | T | G | T | G | G | G | G | G | a | a | a | a | a | a | a | a | 0 |
| F1N214 | α | + | - | A | A | C | A | A | A | A | A | a | a | a | a | a | a | a | b | 1 |
| F1N215 | α | + | - | A | A | C | A | A | A | A | A | b | a | a | a | a | b | b | b | 2 |
| F1N216 | **a** | + | - | T | G | T | G | G | G | G | G | b | a | a | b | b | b | b | a | 3 |
| F1N217 | **a** | + | - | T | G | T | G | G | G | G | G | a | a | b | b | b | b | b | a | 2 |
| F1N218 | n.a. | + | - | T | G | T | G | G | G | G | G | b | a | a | a | a | b | b | a | 3 |
| F1N219 | α | + | - | A | A | C | A | A | A | A | A | a | a | a | a | b | b | b | b | 1 |
| F1N220 | n.a. | + | - | T | G | T | G | G | G | G | G | b | b | b | b | b | a | b | a | 3 |
| F1N221 | **a** | + | - | T | G | T | G | G | G | G | G | a | a | a | a | a | a | a | a | 0 |
| F1N222 | **a** | + | - | T | G | T | G | G | G | G | G | b | b | b | b | b | a | a | a | 1 |
| F1N223 | **a** | + | - | T | G | T | G | G | G | G | G | a | a | a | a | a | a | a | a | 0 |
| F1N224 | α | + | - | A | A | C | A | A | A | A | A | b | b | b | b | b | b | b | b | 0 |
| F1N225 | α | + | - | A | A | C | A | A | A | A | A | b | b | b | b | b | b | b | b | 0 |
| F1N226 | **a** | + | - | T | G | T | G | G | G | G | G | b | b | b | b | b | b | b | a | 1 |
| F1N227 | α | + | - | A | A | C | A | A | A | A | A | b | a | a | a | a | b | b | b | 2 |
| F1N228 | α | + | - | A | A | C | A | A | A | A | A | b | b | b | b | b | b | b | b | 0 |
| F1N229 | **a** | + | - | T | G | T | G | G | G | G | G | b | a | b | b | b | b | b | a | 3 |
| F1N230 | α | + | - | A | A | C | A | A | A | A | A | b | b | b | b | b | b | b | b | 0 |
| F1N231 | α | + | - | A | A | C | A | A | A | A | A | b | b | b | b | b | b | b | b | 0 |
| F1N232 | α | + | - | A | A | C | A | A | A | A | A | a | a | a | a | a | a | a | b | 1 |
| F1N233 | **a** | + | - | T | G | T | G | G | G | G | G | b | b | b | b | b | a | a | a | 1 |
| F1N234 | α | + | - | A | A | C | A | A | A | A | A | b | b | b | b | b | b | b | b | 0 |
| F1N235 | α | + | - | A | A | C | A | A | A | A | A | b | b | b | b | a | a | a | b | 2 |
| F1N236 | **a** | + | - | T | G | T | G | G | G | G | G | a | a | a | a | a | a | a | a | 0 |
| F1N237 | α | + | - | A | A | C | A | A | A | A | A | a | b | b | b | b | b | a | b | 3 |
| F1N238 | α | + | - | T | G | T | G | G | G | G | G | a | a | a | a | a | a | a | a | 0 |
| F1N239 | α | + | - | A | A | C | A | A | A | A | A | b | b | b | a | a | a | a | b | 2 |
| F1N240 | α | + | - | A | A | C | A | A | A | A | A | a | a | a | b | b | b | b | b | 1 |
| F1N241 | α | + | - | A | A | C | A | A | A | A | A | a | a | a | a | a | a | a | b | 1 |
| F1N242 | α | + | - | A | A | C | A | A | A | A | A | a | a | a | a | a | a | a | b | 1 |
| F1N243 | α | + | - | A | A | C | A | A | A | A | A | b | b | b | b | b | b | b | b | 0 |
| F1N244 | α | + | - | A | A | C | A | A | A | A | A | a | a | a | a | a | b | a | b | 3 |
| F1N245 | α | + | - | A | A | C | A | A | A | A | A | b | b | a | a | a | a | b | b | 2 |
| F1N246 | **a** | + | - | T | G | T | G | G | G | G | G | b | b | b | b | a | a | a | b | 2 |
| F1N247 | **a** | + | - | T | G | T | G | G | G | G | G | a | a | a | a | a | a | a | a | 0 |
| F1N248 | α | + | - | A | A | C | A | A | A | A | A | a | b | b | b | b | b | b | b | 1 |
| F1N249 | α | + | - | A | A | C | A | A | A | A | A | a | b | b | b | b | b | b | a | 2 |
| F1N250 | **a** | + | - | T | G | T | G | G | G | G | G | a | a | a | a | a | a | a | b | 1 |
| F1N251 | α | + | - | A | A | C | A | A | G | G | A | a | a | a | a | a | a | a | b | 1 |
| F1N252 | α | + | - | A | A | C | A | A | A | A | A | b | b | b | b | b | b | b | b | 0 |
| F1N253 | **a** | + | - | T | G | T | G | G | G | G | G | a | a | a | a | a | a | a | a | 0 |
| F1N254 | α | + | - | A | A | C | A | A | A | A | A | a | b | b | b | b | b | b | a | 2 |
| F1N255 | **a** | + | - | T | G | T | G | G | G | G | G | a | a | a | a | a | a | a | a | 0 |
| F1N256 | n.a. | + | - | A | A | C | A | A | A | A | A | a | b | b | b | b | b | b | b | 1 |
| F1N257 | **a** | + | - | T | G | T | G | G | G | G | G | a | a | a | a | a | a | a | b | 1 |
| F1N258 | α | + | - | A | A | C | A | A | A | A | A | b | b | b | b | a | b | b | a | 3 |
| F1N259 | α | + | - | A | A | C | A | A | A | A | A | b | b | a | a | a | b | b | a | 3 |
| F1N260 | α | + | - | A | A | C | A | A | A | A | A | a | a | a | a | a | b | b | b | 1 |

The yellow filling color highlights the only *ade2 URA5* progeny, F1N072; the blue filling color highlights the nucleotides that underwent gene conversion in the two progeny, F1N044 and F1N251.

^a^: Mating types were determined based on mating assay by backcrossing each progeny to the two parental strains. “**a**” indicates *MAT***a** progeny; “α” indicates *MAT*α progeny; “n.a.” indicates the progeny did not mate with either parent (i.e. the progeny is sterile).

^b^: “+” indicates wild type; “-“ indicates auxtrophic;

^c^: polymorphic sites located within the *RPO41* gene;

^d^: polymorphic sites located within the inter-genic region between the *RPO41* and *BSP2* genes;

^e^: polymorphic sites located within the *BSP2* gene;

^f^: PCR-RFLP markers, see Table S1;

^g^: PCR markers, see Table S1;

^h^: “F1” indicates the strain was isolated from the F1 generation of the laboratory crosses between JEC169 and S13; “N001” indicates the strain is the progeny No.1 in the series.
